# Are genetic variation and demographic performance linked?

**DOI:** 10.1111/eva.13487

**Published:** 2022-10-04

**Authors:** Lauren N. Carley, William F. Morris, Roberta Walsh, Donna Riebe, Tom Mitchell‐Olds

**Affiliations:** ^1^ University Program in Ecology Duke University Durham North Carolina USA; ^2^ Biology Department Duke University Durham North Carolina USA; ^3^ Department of Plant and Microbial Biology University of Minnesota Twin Cities St. Paul Minnesota USA; ^4^ Division of Biological Sciences University of Montana Missoula Montana USA

**Keywords:** *Boechera fecunda*, conservation genetics, inbreeding depression, integral projection model, population genetics, population viability

## Abstract

Quantifying relationships between genetic variation and population viability is important from both basic biological and applied conservation perspectives, yet few populations have been monitored with both long‐term demographic and population genetics approaches. To empirically test whether and how genetic variation and population dynamics are related, we present one such paired approach. First, we use eight years of historical demographic data from five populations of *Boechera fecunda* (Brassicaceae), a rare, self‐compatible perennial plant endemic to Montana, USA, and use integral projection models to estimate the stochastic population growth rate (*λ*_S_) and extinction risk of each population. We then combine these demographic estimates with previously published metrics of genetic variation in the same populations to test whether genetic diversity within populations is linked to demographic performance. Our results show that in this predominantly inbred species, standing genetic variation and demography are weakly positively correlated. However, the inbreeding coefficient was not strongly correlated with demographic performance, suggesting that more inbred populations are not necessarily less viable or at higher extinction risk than less inbred populations. A contemporary re‐census of these populations revealed that neither genetic nor demographic parameters were consistently strong predictors of current population density, although populations showing lower probabilities of extinction in demographic models had larger population sizes at present. In the absence of evidence for inbreeding depression decreasing population viability in this species, we recommend conservation of distinct, potentially locally adapted populations of *B. fecunda* rather than alternatives such as translocations or reintroductions.

## 1 INTRODUCTION

As human impacts cause the number of declining and threatened populations to increase over time (Pimm et al., 2014), elucidating the factors that predict the persistence and viability of populations is of critical importance. Rare and threatened species often face a suite of potential demographic challenges, including reduced connectivity and mate availability (Aguilar et al., 2006; Delnevo et al., 2020; Gascoigne et al., 2009; Stevens et al., 2018) and increased vulnerability to stochastic demographic and environmental events (Fagan & Holmes, 2006; Gilpin & Soulé, 1986), all of which may influence population viability. Simultaneously, decreased population size and limited connectivity often cause genetic variation to decline in rare and threatened populations (Lande, 1988). Reduced genetic variation can limit a population's ability to respond to natural selection (Lande & Shannon, 1996; Storfer, 1996) and can also reduce the fitness of individuals in the population through the expression of inbreeding depression (hereafter, “ID”). As such, surveying genetic variation in rare and threatened populations is becoming an increasingly common approach to assessing the integrity of populations (Ouborg et al., 2010; Schemske et al., 1994). While there is evidence that genetic variation is correlated with attributes such as population size (Frankham, 1996; Leimu et al., 2006), it is still not clear whether and when genetic surveys can provide useful information regarding the future fates of populations. There is an intuitive sense that population genetics and demographic processes should be correlated—specifically, that low genetic diversity can increase extinction risk. This expectation has been verified experimentally in certain taxa (Newman & Pilson, 1997; Saccheri et al., 1998), but overall, few studies have directly characterized this relationship.

Because genetic diversity and demographic processes can mutually influence and sometimes exacerbate one another (e.g., in “extinction vortices”; Gilpin & Soulé, 1986, Lynch et al., 1995), teasing apart their effects on population viability has been an enduring goal in conservation biology (Richards et al., 2003; Wootton & Pfister, 2013). Inbreeding depression, which is widespread (Charlesworth & Charlesworth, 1987; Jiménez et al., 1994; Keller & Waller, 2002; Nieminen et al., 2001), is one mechanism that may link genetic variation to population persistence or decline. Because ID occurs when reduced heterozygosity exposes deleterious effects of recessive alleles, small or declining populations are expected to show increased ID as the likelihood of mating with close relatives (or within the same individual, in the case of self‐compatible plants) increases. Indeed, some populations showing higher rates of inbreeding have been found to decline in size over time and suffer heightened risk of extinction (Hartfield & Glémin, 2016; Takebayashi & Morrell, 2001). However, generalizable predictions about the impacts of inbreeding on individuals and populations are difficult to make; a suite of biological phenomena may modify the extent to which increased homozygosity reduces fitness. Notably, expression of ID may vary depending on the history of inbreeding in a species due to purging, in which natural selection removes deleterious recessive alleles from populations and thus attenuates the effects of inbreeding over time (Byers & Waller, 1999; Charlesworth & Willis, 2009; Pekkala et al., 2014; Swindell & Bouzat, 2006a, 2006b; Weller et al., 2005). Empirical support for the occurrence of purging in inbred populations is mixed, however, and ID is often still detectable in species that are naturally highly inbred (Byers & Waller, 1999; Carr & Dudash, 2003; Charlesworth & Willis, 2009; Crnokrak & Barrett, 2002; Winn et al., 2011).

Other factors further muddy straightforward predictions about the effects of ID on fitness and population viability. The genetic architecture of ID, which may vary across species, influences whether and when purging is likely to be effective; for example, purging may be more likely to occur when ID is caused by strongly deleterious recessive alleles, while smaller‐effect loci may be more difficult to purge (Charlesworth & Willis, 2009; Coyne & Orr, 2004). Thus, ID may be prevalent even in populations that are naturally highly inbred (e.g., self‐pollinating plants) if it is caused by many small‐effect loci, but may be minimal or absent in highly inbred populations if it is initially caused by a few loci of large effect. The efficacy of selection also varies with effective population size (Charlesworth, 2009), which is often negatively correlated with the rate of inbreeding (Charlesworth & Wright, 2001). This can cause the accumulation of genetic load in inbred populations, driven by the fixation of weakly deleterious mutations that would otherwise be selected against in outcrossing populations. Further, the extent to which ID is expressed varies across environmental conditions; for example, negative effects of inbreeding may be offset by reproductive assurance when mates are scarce, shifting the cost of inbreeding to a net fitness benefit (Barner et al., 2011; Moeller & Geber, 2005), and stressful environments may amplify (Cheptou & Donohue, 2011) or mask (van Etten et al., 2021) ID. Even within a given environment, ID may differ across the life cycle, tending to be more strongly expressed in later‐life fitness components (Angeloni et al., 2014). Finally, the relationship between inbreeding and fitness can vary across genotypes or families within populations (Angeloni et al., 2011), even sometimes changing in sign (Ågren & Schemske, 1993).

Thus, while the intuitive notion that genetic variation influences population persistence may be true in many circumstances, a suite of other factors including evolutionary history (e.g., presence and efficacy of past purging, population size and ability to respond to selection, genetic architecture of ID, etc.), mating system (e.g., self‐compatibility, the rate of inbreeding vs. outcrossing), and environmental context makes the magnitude and direction of these relationships difficult to predict (Table 1). The complexity of interactions among these different processes has caused some authors to suggest it may be “impossible to make universal predictions” about the consequences of inbreeding on organisms and populations (Byers & Waller, 1999). The under‐representation of selfing and mixed‐mating species in conservation genetic research (e.g., only 22 of 156 studies included in Frankham, 2015) exacerbates this problem. As such, many ambiguities remain in our understanding of the relationship between genetic variation, fitness, and population viability. Identifying appropriate conservation and management strategies hinges upon understanding the contributions of these various phenomena to population viability given the biology of the population(s) of concern. For example, if ID is prevalent in a population or species, transplanting or relocating individuals across populations may increase mate availability, outcrossing, and fitness (Luijten et al., 2002). Conversely, if ID is weakly expressed or nonexistent, transplants or translocations may convey little benefit to population viability, or may even cause harm if outcrossing disrupts combinations of locally adapted alleles (Edmands, 2007).

**TABLE 1:** A nonexhaustive overview of demonstrated and putative links between genetic variation and either individual fitness or population performance, depending on a population's evolutionary history.

History	Metric	Direction of effect	Example(s)
“Standard”	Individual fitness	Positive	Meta‐analytic evidence for prevalence of inbreeding depression in plants (Angeloni et al., 2014), wild animals (Vega‐Trejo et al., 2022), and livestock (Leroy, 2014); heterosis in crop plants (Hochholdinger & Baldauf, 2018)
	Population performance	Positive	Decreased *λ* with increased inbreeding (Bozzuto et al., 2019); increased probability of extinction with low effective population size (Newman & Pilson, 1997); correlations between λ and allelic richness (Hens et al., 2017)
“Alternative”	Individual fitness	Positive	Persistence of some inbreeding depression in selfing plants (Winn et al., 2011)
		Weak or no effect	Reduced inbreeding depression in fruit fly lineages with history of purging (Swindell & Bouzat, 2006a, 2006b); reduced benefits of genetic rescue in selfing and mixed‐mating species compared with self‐incompatible and outcrossing species (Frankham, 2015)
		Negative	Outbreeding depression expressed in among‐population *Arabidopsis* crosses (Oakley et al., 2015) and selfed morning glories (van Etten et al., 2021)
	Population performance	Positive	Few empirical tests
		Weak or no effect	Effects of inbreeding depression on fitness components but not *λ* (Johnson et al., 2011)
		Negative	Few empirical tests; evidence from simulations that removal of deleterious mutations by purging can dampen extinction risk (Caballero et al., 2017)

*Note*: Under a “standard” evolutionary history, such as a predominantly outcrossing mating system, the general expectation is that genetic variation is positively correlated with individual fitness and population performance. Conversely, under “alternative” evolutionary histories (e.g., a history of purging of deleterious recessive alleles; selfing reproductive mode, etc.), genetic variation may be weakly or even negatively correlated with individual fitness. Thus, scaling up expectations from individuals to populations is less straightforward. Direct tests of the relationships between genetic variation and population performance in systems with “alternative” evolutionary histories are scarce.

Despite the many plausible biological links between genetic variation and population viability, most studies assessing the fitness effects of genetic variation and ID assess fitness components (e.g., survival, reproduction) rather than integrated metrics (e.g., lifetime fitness, population growth rates) (Angeloni et al., 2014). However, meta‐analysis has revealed that the effects of ID often vary across different fitness components and/or life history stages, tending to be more strongly expressed in reproductive traits expressed later in life (Angeloni et al., 2014). Because of this, approaches that fail to track long‐lived individuals across the life cycle may be insufficient to robustly test for the effects of genetic variation on lifetime fitness and, consequently, population viability. Comparatively few studies have interrogated the relationships between population genetics and population dynamics directly, in part because collection of complementary genetic and demographic data is uncommon (Bozzuto et al., 2019; Freitas et al., 2020; Heschel & Paige, 1995; Richards et al., 2003). Further, when such relationships are studied, proxy measurements for genetic variation (e.g., population size [Fischer & Matthies, 1998; Menges, 1991]) and/or demographic performance (e.g., individual fitness components [Heschel & Paige, 1995; Jiménez et al., 1994; Menges; 1991; Oostermeijer et al., 1994], population size [van Treuren et al., 1991]) are often used instead of more directly relevant metrics such as quantitative estimates of standing genetic variation and long‐term population growth rates. Finally, even those studies that do explicitly characterize both genetic and demographic patterns across populations rarely assess the relationships among these metrics, treating them instead as separate lines of evidence to consider when making management decisions (Schemske et al., 1994) or comparing the outcomes of experiments or demographic simulations manipulating genetic variation categorically, at coarse scales (e.g., increased vs. decreased ID [Johnson et al., 2011]; with or without translocations from genetically diverse populations [Madsen et al., 1999; Westemeier et al., 1998]). Thus, while together these studies suggest that genetic variation may indeed often benefit populations (Dudash & Fenster, 2000), there are still few studies that have directly characterized relationships between genetic variation and population viability (but see Bozzuto et al., 2019; Endels et al., 2007; Hens et al., 2017; Newman & Pilson, 1997, Saccheri et al., 1998). In an era in which sequencing technology is inexpensive and demographic censusing remains costly and laborious, it is also prudent to ask whether genetic metrics (Teixeira & Huber, 2021), demographic metrics (Crone et al., 2012), or both can provide useful information about the future fate of populations of conservation concern. Long‐term research combining demographic and genetic approaches (e.g., Bozzuto et al., 2019; Hens et al., 2017) is uniquely poised to address this important question.

Here, we leverage one such dataset to answer two key questions related to these knowledge gaps in the conservation genetics literature:

1. Are previously posited positive relationships between genetic variation and population dynamics generalizable to species with “alternative” evolutionary histories, e.g., a predominantly selfing reproductive mode?
2. When both genetic and demographic data are available, which better predict the fates of at‐risk populations?

Specifically, we combine long‐term field data with genetic surveys to empirically assess potential links between genetic variation and population viability. We present eight years of demographic data collected in the 1990s spanning 17,994 individuals across five populations of the self‐compatible perennial herb *Boechera fecunda* (Brassicaceae), a rare and threatened species endemic to southwestern Montana, USA (Rollins, 1993). Because mixed‐mating systems in plants often yield variable outcrossing rates and levels of standing genetic variation across populations (Ruane et al., 2020), we leveraged previously described metrics of genetic variation and inbreeding across the monitored populations (Song & Mitchell‐Olds, 2007) to explicitly test whether population genetic parameters are correlated with long‐term stochastic population growth rates, extinction risk, and predicted contemporary population size. Finally, by revisiting the historical census sites two decades later, we tested whether and which demographic and/or population genetic parameters predicted contemporary population persistence and population density.

## 2 METHODS

### 2.1 Study species

**FIGURE 1:**
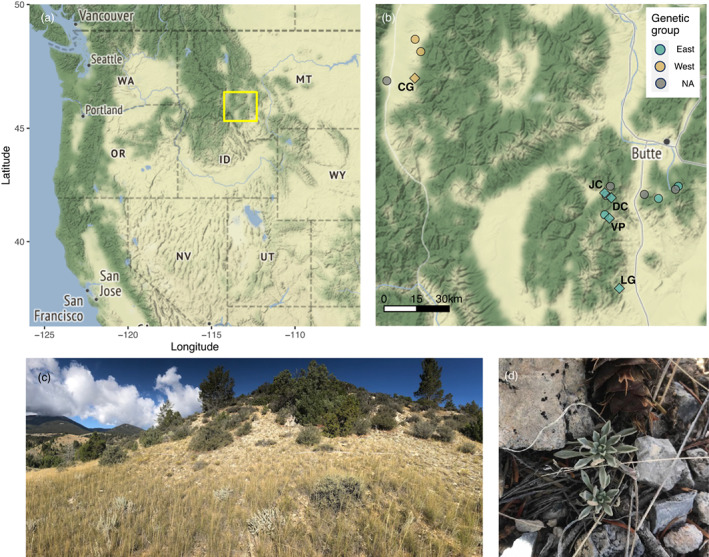
The focal species, *Boechera fecunda*, and its habitat. (a) *B. fecunda* is endemic to a small region in southwestern Montana, USA (gold box). (b) In the focal region, 21 populations have been previously described (Lesica & Young, 2005). The 15 known populations for which location data have been previously published (Lesica & Young, 2005; Song & Mitchell‐Olds, 2007) are shown as points on the map. Each population's genetic group, if known (Song & Mitchell‐Olds, 2007) is shown by the point color. The five populations that were censused in this study are marked with diamonds and the site ID, and other surrounding populations are marked with circles. (c) Typical *B. fecunda* habitat as observed at the Lime Gulch site in September 2019. (d) Two *B. fecunda* rosettes found during the 2019 recensus at Lime Gulch.

*Boechera fecunda* (formerly *Arabis fecunda*: Rollins, 1984; Brassicaceae) is a rare perennial herb extant in only 21 populations in southwestern Montana (Figure 1; Rollins, 1993). It is classified as a sensitive species by the US Forest Service and Bureau of Land Management, a species of concern in the state of Montana (Montana Natural Heritage Program, 2021), and a candidate for listing as a threatened species by the US Fish and Wildlife Service (USDI‐FWS, 1993). *B. fecunda* grows on rocky calcareous and/or cryptogamic soils (Lesica, 1993), which may contribute to the species' limited distribution. Calcareous soils are sandy in texture and contain little organic matter, making them subject to erosion on the steep slopes upon which *B. fecunda* typically grows (Lesica, 1993), and cryptogamic soils (living biological crusts containing algae, cyanobacteria, bryophytes, and fungi) develop slowly and are easily damaged (Lesica & Shelly, 1992). Both of these habitat types are easily disturbed, and although direct herbivory to *B. fecunda* is uncommon, grazing livestock may trample critical habitat and exacerbate erosion and habitat loss (Montana Natural Heritage Program, 2021).

Livestock trampling and other disturbance events such as road construction and mining also promote the spread of invasive species in this region, including spotted knapweed (*Centaurea maculosa* [Asteraceae]). In undisturbed communities, competition between *B. fecunda* and heterospecific neighbors appears to be weak (Lesica, 1993), as other species struggle to recruit into the dry, nutrient‐poor soils where *B. fecunda* grows. Spotted knapweed, however, thrives in these habitats and has been shown to reduce establishment rates of *B. fecunda* (Lesica & Shelly, 1996). Thus, natural and anthropogenic disturbances may threaten *B. fecunda* populations both directly, through habitat destruction, and indirectly, by facilitating the spread of competitors.

### 2.2 Demographic monitoring

We focused on five of the 21 known *B. fecunda* populations to monitor demographic processes in this rare species (Figure 1b). Most populations are separated by tens of kilometers, and the closest pair of focal populations (DC and JC) are ~4 km apart. Due to the species' rarity, the study region was thoroughly searched for extant populations at the outset of the study period (T. Mitchell‐Olds, *pers. obs*.), and it is unlikely that there are undetected populations nearby. This disjunct distribution, combined with the species' predominantly self‐pollinating reproductive mode (i.e., flowers are seldom visited by pollinators) and gravity‐dispersed seeds, makes it unlikely that these populations are connected by dispersal or gene flow.

In 1990, transects were established spanning each of the five focal populations, and 1 m × 1 m quadrats were laid along each transect. The five focal populations were selected because they span the majority of the elevational range of the species, and also because they captured apparent variation in population size and vigor at the outset of the study (Walsh, 1992). Because of variability in population size, the number of transects and quadrats varied across populations, ranging from 1 to 3 transects and 7 to 45 quadrats per population. Within quadrats, each individual plant was tagged and identified using X and Y coordinates to designate its location. At the outset of the study, ~500 individuals were tagged in each population except for Jerry Creek, where only ~300 individuals were present (Walsh, 1992). From 1990 to 1997 (seven annual transitions), the populations were visited annually and censused to measure the size (rosette diameter), survival (0/1), and fecundity (number of fruits) of marked individuals. New recruits were counted each year and tagged for censusing in future years.

### 2.3 Life‐history details

In this species, most plants progress through the life cycle as follows: (1) seeds germinate and establish as a vegetative basal rosette, (2) vegetative rosettes may or may not bolt, i.e., produce a flowering stalk, (3) flowering stalks may produce fruits, which bear seeds and start the life cycle again (Figure S1A). Bolting is a necessary prerequisite for reproduction, but not all rosettes bolt in every year. Furthermore, across the perennial life span, individuals may be either iteroparous (i.e., bolt and reproduce multiple times across years) or semelparous (i.e., bolt and reproduce only once before dying), and these alternative life‐history strategies may exhibit demographic trade‐offs with survival (Lesica & Young, 2005). Bolting stalks senesce following reproduction, and new ones emerge from the basal rosette in future years for individuals that are iteroparous. Bolting stalks typically grow from axillary buds among the rosette leaves (Lesica & Shelly, 1995). The basal rosette may grow or shrink in size between years.

In addition to these “normal” life‐history transitions, some *B. fecunda* individuals may produce a bolting stalk from the terminal bud at the center of the rosette (Lesica & Shelly, 1995). Often, this pattern of reproduction occurs in individuals in which the leaves of the basal rosette have died back or are missing. Reproduction via the terminal bud has been previously described as fatal (Lesica & Shelly, 1995), although we found this is not always the case. Here, we call individuals displaying this strategy “bolters”, since they typically have a bolting stalk with no rosette (Figure S1B). It is possible for an individual to reproduce “normally” (i.e., via the axillary buds) in some years and later become a bolter (i.e., reproducing via the terminal bud, sometimes—but not always—followed by death).

Finally, plants may occasionally die back aboveground and then resprout new rosettes from the root stock in future years. We call this state “aboveground dormancy”, meaning that all aboveground tissue has died back, but the individual is still alive (Figure S1C). Because these plants are difficult to detect during censuses, “dormant” individuals were inferred during data analysis from sequences of survival data (details below). Because they lack all aboveground structures, dormant individuals have no measurable size and cannot reproduce until they re‐emerge as either a “normal” plant or as a bolter.

### 2.4 Demographic modeling

#### 2.4.1 Overview of the approach

Because *B. fecunda* is a perennial species with continuous variation in rosette size and (often) iteroparous reproduction, we used integral projection models (IPMs) to model population dynamics. In an IPM framework, vital rates of growth, survival, fecundity, and recruitment are treated as functions of a continuous size variable (Ellner & Rees, 2006; Merow et al., 2014), and population growth is modeled as a function of the continuous size distribution of individuals in the population. In long‐lived species without obvious discrete age or stage classes, this approach avoids arbitrary classification into artificial size bins, which would be required in matrix projection models (MPMs), and often requires fewer model parameters be estimated. Here, we modeled size‐dependent vital rates using rosette diameter as the independent variable.

While mainly focusing on continuous size‐dependent vital rates as the drivers of population dynamics, we structured our IPMs in a multimatrix framework to also account for transitions between discrete life‐history states (“normal”, dormant, and bolter, as described above) and potential changes in vital rate functions across states. In general, we constructed a multimatrix structure (similar to Berry et al., 2008) such that each discretized IPM kernel contained 9 submatrices representing transitions among the three discrete life cycle states, each containing 150 *×* 150 mesh points spanning sizes from 0 mm to 110% of the largest observed rosette diameter (Figure S2A). We applied different state‐specific survival, growth, reproduction, and recruitment functions to each submatrix, when applicable (Figure S2B).

#### 2.4.2 Vital rate regressions

In all vital rate regressions, unless otherwise noted, we began by fitting a global mixed‐effects model (‘lme4’ package in R; Bates et al., 2015) including a linear fixed effect of size, a fixed effect of size squared, and random intercept effects of year and quadrat nested within transect. We then performed model selection comparing the global model to nested reduced models containing only a linear effect of size, only a squared effect of size, or no effect of size (intercept‐only), retaining the same random effects across all models. We compared model fit using AICc scores (‘MuMIN’ package in R; Bartón, 2020) and obtained model outputs (regression coefficient estimates, variance–covariance matrices, etc.) from the model with the lowest AICc score, unless two or more models had AICc scores differing by <2, in which case we chose the most parsimonious model containing fewest effects.

We fit vital rate models and performed model selection separately for each of the five censused populations, allowing the shapes of the functions and the parameters retained in best‐fit models to differ across populations. We chose to model populations separately rather than, e.g., including population as an effect in one larger model, for several reasons. First, sample size was sufficient (*N* = 17,994) to give good statistical power to estimate vital rates even after partitioning the full dataset into population‐specific subsets. Second, with only five populations, it is a somewhat arbitrary decision whether a population effect in a larger model should be treated as a fixed or a random effect if included in a model. Third, inclusion of population as an effect (either fixed or random) in vital rate models raises the possibility that population‐by‐year, population‐by‐size, and higher order interactions may also be relevant to include; however, interpretation of such effects can be difficult, and can result in spurious overfitting of vital rate models. Fourth, because we were predominantly interested in looking at continuous patterns in demographic trends across populations, not, e.g., comparing means across categories, there was no need to test the “significance” of population on vital rates or demographic outcomes. Finally, fitting vital rate regressions separately allows for the estimation of the shapes of size‐based relationships to vary independently across populations, free from any assumptions constraining parameter estimation in a larger model.

Across the five populations and including specific models for different discrete states, we modeled 77 vital rates (Table S1). Detailed methods describing the specific approaches used for each of these models are provided in Appendix A.

#### 2.4.3 Discrete life‐history transitions

As described above (see “Life‐history details”), *B. fecunda* individuals can transition between three discrete life‐history states. Two of these, dormant individuals and bolters, have no aboveground size. Because of this, these states needed to be handled differently in a continuous size‐based IPM framework (Appendix A). In addition, classification of individuals into discrete state spaces from the underlying patterns in the census data was necessary.

Because dormant individuals have no aboveground tissue (Figure S1C), they are most often marked as dead during censuses. As such, 6.5% of individuals in the demographic data (1177 of 17,994 total unique plants observed between 1990 and 1997) showed patterns of apparent “reincarnation”, i.e., were marked as alive both before and after a census at which they were presumed dead. To prevent individuals emerging from dormancy from being counted as new seedlings, which would inflate estimates of recruitment, we revised the survival records of apparent reincarnates such that intermediate “deaths” flanked by observations of a living plant were corrected. For example, an individual with an 8‐year survival history of “00011101” would be corrected to “00011111”. We then scored dormancy across census years as a derived variable by comparing the original and corrected survival records (i.e., in the previous example, the individual would be marked dormant in the seventh year and in no other years).

Similarly, 10% of individuals (1850 of 17,994) were bolters in at least one census year. To avoid overestimation of shrinkage to 0 or small sizes in our growth models, we treated bolters as a separate discrete state. We identified bolters in the dataset based on the criteria that survival was scored as 1 and rosette diameter was scored as 0 or NA.

In this long‐term dataset, we found that bolters may become dormant aboveground and dormant plants may become bolters. As such, in addition to fitting separate vital rate regressions for each state, we modeled the probability of transitioning between bolter, dormant, and “normal” states, making these transitions size‐dependent if warranted (Figure S2; Appendix A).

#### 2.4.4 Interannual variation and parameter uncertainty

For each population, we constructed a series of multimatrix IPM kernels to calculate the long‐term stochastic population growth rate (*λ*_S_) and to project population sizes. Specifically, we built one multimatrix for each intercensus interval, adding annual deviations to the overall model intercept by sampling from the Normal distribution of the random year coefficient, incorporating parameter uncertainty in the random effect variance (Appendix A; Dennis et al., 1991). When calculating each sampled multimatrix, we also accounted for uncertainty in the estimation of parameters describing the effects of size on each vital rate. To do so, we used parametric bootstrap sampling from the multivariate Normal distribution defined by the best estimates of the regression coefficients and the variance–covariance matrix of the fixed effects of each final vital rate regression following model selection (as in Visser et al., 2018; Wilbur et al., 2016, etc.).

#### 2.4.5 Asymptotic dynamics

To describe long‐term population dynamics, we computed the stochastic population growth rate (*λ*_S_) of each of the 5 censused populations. For each bootstrap sample of the vital rate regression coefficients, we calculated the set of seven annual multimatrix projection kernels as described above. Beginning with population vector representing a uniform size distribution (Appendix A), we projected the vector forward 50,000 times by multiplying it by one of the seven kernels, randomly chosen at each time step (thus assuming that environmental states are independently and identically distributed). At each projection step, we calculated an annual λ as the sum of the size vector at time *t* + 1 divided by the sum of the size vector at time *t*. We renormalized the sum of the resultant vector to 1 after each projection step to avoid rounding errors (Caswell, 2001). We then estimated *λ*_S_ as the geometric mean of the 50,000 λ estimates in the sequence. We repeated this process 5000 times, resampling coefficients for fixed effects and the random effect of year used in vital rate functions in each bootstrap run. We used the resulting 5000 bootstrapped values to estimate the distribution of *λ*_S_ for each population.

#### 2.4.6 Transient projections and population viability

In addition to *λ*_S_, we projected population sizes from the final census year (1997) to 2019. For projections, we scaled the starting size vector (Appendix A) to the empirical population size in the final census year, and projected the resultant vector forward through time using random draws of sampled annual matrices as described above. Within each bootstrap run, we ran 1000 projections for each population using different 22‐year random sequences of annual kernels (Figure S3). Across these 1000 replicate projections, we obtained the median projected population size at each future year (1998–2019) as well as the proportion of projections ending in extinction. (We used the median because population size is typically positively skewed, making the mean a poor indicator of what most trajectories do [Lewontin & Cohen, 1969]). Because *B. fecunda* flowers are hermaphroditic and self‐compatible, populations of only one individual may theoretically persist, so we defined the local extinction threshold as zero individuals. (We rounded predicted population sizes to the nearest integer at each step during transient projections, so extinction to zero was always possible.) We calculated the mean of the predicted population size in 2019 (Pred[*N*_2019_]) as the grand mean of the 5000 bootstrapped mean population size predictions. Similarly, we calculated the mean probability of local extinction (*Pr*_ext_) from the 5000 bootstrapped estimates of the proportion of population trajectories ending in extinction. We calculated 95% confidence intervals for both of these metrics from the distributions of bootstrapped values.

### 2.5 Assessing relationships between genetics and demography

#### 2.5.1 Molecular genetic data

*Boechera fecunda* is closely related to other model organisms in the family Brassicaceae, including *Arabidopsis thaliana* and *Boechera stricta*; as such, it is amenable to study using molecular genetic techniques (Rushworth et al., 2011). A previous study investigated genetic variation in ten *B. fecunda* populations, including our five focal demographic populations, utilizing 13 neutral microsatellite loci and 27 single nucleotide polymorphisms (Song & Mitchell‐Olds, 2007). Seven metrics of genetic diversity, including polymorphism, heterozygosity, allelic richness, and the inbreeding coefficient (*F*_IS_), were estimated from these data (Table 2). These population genetic parameters reflect genetic variation present in the populations during the same time period in which demographic data were collected in the field; all seeds used for molecular genetic analysis in Song and Mitchell‐Olds (2007) were collected before 1998 (T. Mitchell‐Olds, *pers. comm*.). Many of these genetic metrics capture similar information, and, as such, are highly correlated across populations (Figure S4). Because of this, we chose two genetic variables to focus on in our analyses: *H*_O_ and *F*_IS_.

**TABLE 2:** Population genetic parameters used in these analyses (*sensu* Song & Mitchell‐Olds, 2007)

Population	*P*	*n*_a_	*n*_e_	*H*_O_	*H*_S_	*R*_S_	*F*_IS_
CG	92.3	3.31	2.4	0.102	0.54	3.21	0.81
JC	7.7	1.08	1.05	0.006	0.03	1.08	0.81
DC	46.2	1.92	1.40	0.043	0.20	1.83	0.78
VP	30.8	1.31	1.13	0.030	0.09	1.30	0.66
LG	15.4	1.15	1.11	0.008	0.07	1.15	0.89

Abbreviations: *P*, percent polymorphic loci; *n*_a_, observed allele number; *n*_e_, effective allele number; *H*_O_, observed heterozygosity; *H*_S_, gene diversity; *R*_S_, allelic richness; *F*_IS_, inbreeding coefficient.

*H*_O_, the observed level of heterozygosity, captures information about the proportion of individuals in a given sample that are heterozygous at the focal loci; in the 5 focal *B. fecunda* populations, this metric is highly correlated with *P* (percent polymorphic loci), *n*_a_ (observed allele number), *n*_e_ (effective allele number), *H*_s_ (gene diversity) and *R*_s_ (allelic richness) (*r* = 0.97–1.00; Figure S4). Conversely, *F*_IS_, the inbreeding coefficient, is only weakly correlated with *H*_O_ and the other five genetic parameters. This is because, while the other metrics describe the genetic variation present in a population, *F*_IS_ describes the arrangement of that genetic diversity within and among individuals (i.e., the deficit of heterozygotes relative to Hardy–Weinberg expectations), which is influenced by inbreeding and/or population structure (Hartl & Clark, 2007). While *H*_O_ captures general patterns of standing genetic variation across these populations, the inbreeding coefficient is most relevant to the question of how ID might affect population dynamics.

#### 2.5.2 Statistical models

We combined the two focal genetic parameters with the outcomes of our demographic analyses described above to test whether population genetic characteristics predict population performance. Because several of these metrics are bounded by 0 and/or 1, we transformed estimates to test correlations on a linear scale. Specifically, we transformed *λ*_s_ and Pred(*N*_2019_) on the natural log scale and *P*_e_, *H*_O_, and *F*_IS_ on the logit scale. We adjusted all values of Pred(*N*_2019_) and *P*_e_ with a small constant to avoid dropping 0 and 1 values, respectively, during transformation. After transformation, we tested for linear correlations between demographic parameters and both genetic parameters by calculating the Pearson correlation coefficients.

**FIGURE 2:**
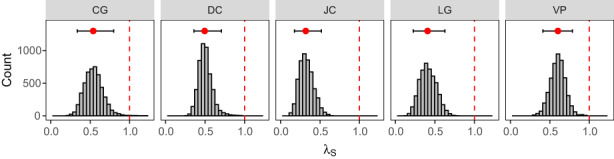
Distribution of stochastic population growth rates calculated from 5000 bootstrapped 50,000‐year projections. Within each panel, the vertical dashed line marks λ = 1 (stable population growth). Red circle shows the median of bootstrapped values, with black horizontal lines showing the range from the 2.5th to 97.5th percentiles of bootstrapped values.

Prior studies have found that there is population structure across the *B. fecunda* range, with two main population groups, East and West, showing divergent evolutionary histories (Song & Mitchell‐Olds, 2007; Figure 2b). East and West groups also differ significantly in ecologically important traits including individual plant growth rate and water use efficiency (Leamy et al., 2014; McKay et al., 2001). Of the five populations for which demographic data were collected, only one (CG) represents the West group; thus, it is not possible to test statistically for group‐level differences in population genetic characteristics, demographic processes, and their relationships. However, in case differences in evolutionary history may have influenced these patterns, we also assessed all correlations described above excluding the CG population, to focus exclusively on the East population group (albeit with only four populations).

### 2.6 Assessing the predictive power of genetic and demographic parameters

In September 2019, at the end of the growing season, we revisited all five focal populations to estimate contemporary population persistence, size, and density. Markers of original transect, quadrat, and individual plant locations were not maintained in the intervening 22 years, so we used a handheld GPS to locate the coordinates marking the approximate center of the historical census sites. We searched in a ~150 m radius of each central point for any *B. fecunda* individuals, and, if discovered, established a new 20 m transect passing through the densest part of the population. We then counted all *B. fecunda* individuals in a 40 m^2^ area spanning 1 m on either side of the transect. Within each 1 m^2^ search area, we divided the total number of living individuals by the search area to estimate the contemporary *B. fecunda* population density in each quadrat. We calculated the mean contemporary density, and 95% confidence intervals around the mean, from the 40 quadrats sampled per site. To determine whether demographic and/or genetic population parameters measured previously predict current population density, we used Pearson correlations to test for relationships between mean population density in 2019 and transformed demographic (*λ*_S_, *Pr*_ext_, and Pred(*N*_2019_)) and genetic (*H*_O_, *F*_IS_) predictors, after transforming density measurements on the natural log scale.

Finally, to more straightforwardly compare the data from the contemporary recensus to historical data and predicted outcomes, we converted the latter from population size to density. First, using the historical data, we divided the census size of each population in each year by the number of 1 m^2^ quadrats utilized at that site. Then, to obtain the average historical density for each population, we took the mean value of these 8 annual density estimates. We also converted demographic predictions into density estimates by dividing mean Pred(*N*_2019_) and the 50th, 2.5th, and 97.5th quantiles of Pred(*N*_2019_) by the number of quadrats utilized at each site to obtain the mean, median, and 95% CI of population density predicted by the demographic simulations.

### 2.7 Statistical software

We performed all data processing, analysis, demographic modeling, and data visualization using R v. 4.0.2 (R Core Team, 2020).

## 3 RESULTS

### 3.1 General demographic patterns

In general, the vital rates differed across discrete life‐history stages. For most vital rates that were modeled as size‐dependent, model selection retained quadratic effects of size in “normal” plants; (Table S1); both survival and growth were highest for mid‐sized individuals but declined for large individuals in almost all populations (Figure S5), perhaps due to aging. In contrast, in the final regression models, the vital rates of bolters and dormant plants were predominantly either linear functions of size or independent of size (Table S1). For example, the probability of reproduction was uniformly high for bolters regardless of their prebolting size, while the probability of reproduction for nonbolters varied depending on size in all five populations (Figure S5). For most vital rates, the shape of vital rate functions in a given discrete state was similar across populations, but the extent of interannual effects varied across populations (Figure S5).

**FIGURE 3:**
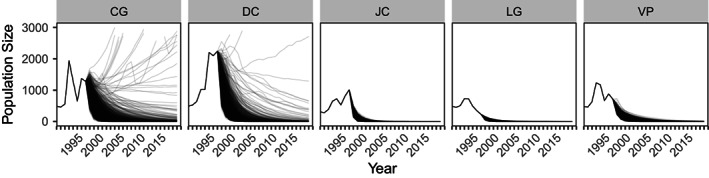
Empirically observed (1990–1997) and projected (post‐1997) population sizes of five *Boechera fecunda* populations. Projections represent median population sizes across 1000 trajectories for each of 5000 bootstrap replicates each population. For clarity, the *y*‐axis ends at 3000, truncating a very small number of trajectories (0.044%) predicting population sizes exceeding *N* = 3000. Examples of replicate trajectories used to derive median values shown here are provided in Figure S3.

Censused population sizes varied substantially among years, with all five populations showing several years of rapid growth and several of severe decline during the survey period. Accordingly, the underlying vital rates also varied among years, with survival rates changing the most through time (Figure S5). Estimates of *λ*_s_ were quite low (median *λ*_s_ = 0.3–0.6; Table 3), in part because of the dampening effect of interannual variation in vital rates on long‐term population growth under IID environmental variation (Tuljapurkar, 1982). Similarly, the majority of bootstrapped trajectories showed likely decline in all five populations (almost all simulations estimating *λ*_s_ < 1; Figure 2). This suggests that, according to the available historical data, population decline was likely to occur in the years following the final census in 1997. Indeed, simulated population projections beginning in 1997 confirmed this (Figure 3), with estimated probability of extinction between 1997 and 2019 exceeding 95%, and median predicted contemporary population size equal to 0, for all five populations (Table 3).

**TABLE 3:** Predicted population dynamics from 1997 to 2019. Stochastic population growth rates (*λ*_S_) were estimated from 5000 bootstrapped replicates of model parameters each with 50,000 time steps

Population	Median *λ*_S_ (95% CI)	Mean probability of extinction (95% CI)	Mean predicted population size in 2019 (95% CI)	Mean predicted population density in 2019 (95% CI) (m^−2^)	Observed mean population density in 2019 (95% CI) (m^−2^)
CG	0.539 (0.337–0.799)	0.901 (0–1)	48.3 (0–9)	3.22 (0–0.6)	0.300 (0–3.0)
JC	0.319 (0.175–0.517)	1.000 (1–1)	0 (0–0)	0 (0–0)	0.725 (0–2.0)
DC	0.494 (0.358–0.709)	0.959 (0.099–1)	31.4 (0–1)	1.65 (0–0.053)	23.825 (0–138.4)
VP	0.602 (0.412–0.785)	0.960 (0.168–1)	0.05 (0–1)	0.002 (0–0.014)	9.325 (0–32.1)
LG	0.407 (0.225–0.626)	0.999 (1–1)	0 (0–0)	0 (0–0)	6.575 (0–23.1)

*Note*: Extinction risk and predicted population size in 2019 were estimated as the grand mean values across 5000 bootstrapped samples, each with 1000 22‐year trajectories using random sequences of annual projection matrices.

### 3.2 Relationships between population genetics and demography

**FIGURE 4:**
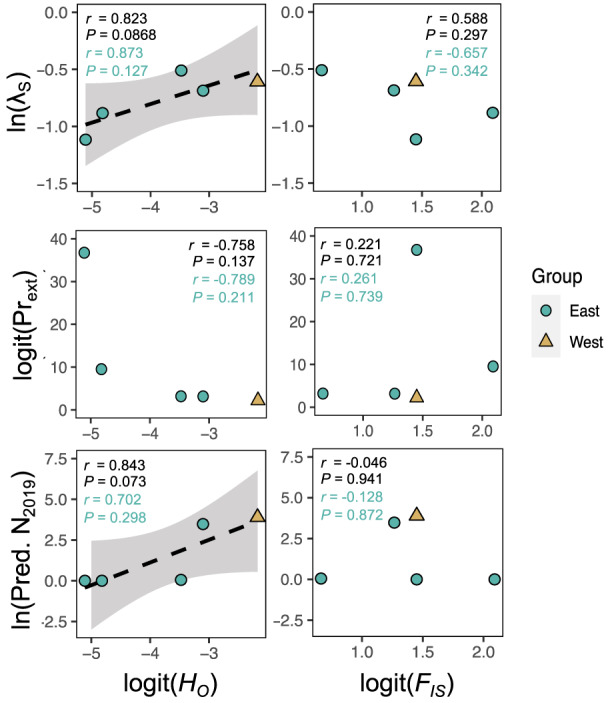
Correlations between population genetic parameters and demographic parameters of five populations of *Boechera fecunda*. Color of points corresponds with population groups (*sensu* Song & Mitchell‐Olds, 2007). Within each panel, text indicates Pearson correlation coefficients and associated *p*‐values for all five populations (black text) or the East population group only (teal text). Dashed black regression lines show marginally significant correlations (*p* < 0.10) across all five populations.

When considering populations across both genetic groups (East and West; Table 2; Song & Mitchell‐Olds, 2007), long‐term stochastic population growth rates and predicted population size were marginally significantly correlated with standing genetic variation (measured by *H*_O_); more genetically diverse populations had higher long‐term growth rates and greater predicted contemporary population sizes (Figure 4, left). The relationship between standing genetic variation and extinction risk also mirrored this pattern, with more genetically diverse populations appearing less likely to go extinct in demographic simulations, although this relationship was not significant. Similarly, the qualitative patterns were the same within the East population group, although none were significant. All correlations between observed heterozygosity and demographic metrics were strong (|*r*| = 0.70–0.87) although the maximum sample size of 5 populations in these regressions limits statistical power.

Conversely, relationships between the inbreeding coefficient *F*_IS_ and demographic outcomes were weaker (|*r*| = 0.05–0.66) and consistently far from statistical significance (Figure 4, right). Populations with a stronger history of inbreeding did not vary substantially in stochastic growth rate, extinction risk, or predicted population size compared with more outbred populations.

### 3.3 Predictive power of demographic and genetic parameters

By recensusing the historical study sites and determining persistence to the present and contemporary population density, we tested the predictive power of both historical demographic and population genetic parameters. Populations of *B. fecunda* were extant at all five study sites from the 1990s, despite model predictions that local extinction was likely (>95%) in all populations (Figure 3; Table 3).

**FIGURE 5:**
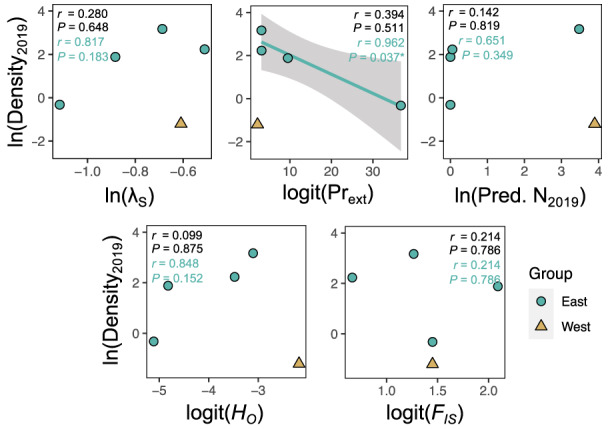
Historical demographic (top) and genetic (bottom) predictors of contemporary population density, estimated in a 2019 recensus. Colors of data points and text as in Figure 4. The solid teal regression line shows a significant correlation (*p* < 0.05) across the four populations in the East group.

Populations with higher predicted extinction risk in demographic models had significantly lower contemporary population densities, albeit only in the East population group (Figure 5, center top). Patterns were qualitatively similar for other demographic metrics among East populations, but were not significant (Figure 5, top). Of the genetic metrics, there was a trend toward populations with higher heterozygosity having higher contemporary population densities, but only in the East population group (Figure 5, bottom left). There was no relationship between inbreeding levels and contemporary density (Figure 5, bottom right). Across all five populations spanning both genetic groups, no demographic or genetic metrics significantly or marginally significantly predicted contemporary population density (Figure 5).

## 4 DISCUSSION

### 4.1 Genetics and demography show mixed relationships in *Boechera fecunda*

We leveraged both long‐term historical demographic data and population genetic parameters to explicitly test for relationships between genetic variation and demography in *Boechera fecunda*, a rare, self‐pollinating Montana endemic plant. We found that in general, correlations in the expected directions exist between standing genetic variation (here, observed heterozygosity) and demographic performance in this species (Figure 4, left). While the statistical significance of these relationships was weak and variable, this suggests potential impacts of genetic variation on population persistence. In this case, more genetically diverse populations were generally predicted by our models to have slightly higher long‐term population growth rates and contemporary population sizes (Figure 4), although significant population declines were predicted overall (Figure 3). This finding is consistent with the idea that greater genetic variation may increase viability in rare and/or fragmented populations. We note that these correlations were strongest when including data from all five populations, and may be influenced by differences in genetic variation across two distinct genetic groups (Song & Mitchell‐Olds, 2007), although the magnitude and direction of relationships is consistent when testing within the East population group only (e.g., for *λ*_S_ ~ *H*_O_, *r*_East+West_ = 0.82 vs *r*_East_ = 0.87).

However, one of the main mechanisms by which low genetic variation is expected to reduce fitness is ID (Bozzuto et al., 2019; Lande, 1988; Szűcs et al., 2014). Among the measurements of genetic variation currently available in this species (Song & Mitchell‐Olds, 2007), the inbreeding coefficient (*F*_IS_) most directly quantifies the level of inbreeding within these populations, and thus is most relevant to the question of how ID might affect population dynamics. While other population genetics statistics describe directly the observed levels of genetic variation in the population, *F*_IS_ describes the deficit of heterozygosity relative to Hardy–Weinberg expectations that inbreeding and/or population structure cause (Hartl & Clark, 2007). In this case, despite the general trends discussed above for *H*_O_ (a proxy for all other available metrics of genetic variation in these populations; Figure S4), we found that *F*_IS_ was not correlated with any demographic metrics obtained from our models. Similar patterns have been observed in other plant species, with genetic variation generally – but not inbreeding rates specifically – correlating with population dynamics (Endels et al., 2007). We thus lack definitive evidence that ID influences population dynamics in *B. fecunda*, despite some evidence for its effects at the individual level in this species (Hamilton & Mitchell‐Olds, 1994) and at the population level in some mammals (Bozzuto et al., 2019). The lack of evidence for ID at the population level could be caused by past purging of deleterious alleles in this predominantly self‐fertilizing species (Byers & Waller, 1999; Charlesworth & Willis, 2009), insensitivity of population dynamics to the particular fitness components exhibiting ID at the individual level (de Kroon et al., 2000; Ehrlén, 2003; Li et al., 2016), and/or limited statistical power to detect a true effect at the population level.

### 4.2 Demographic models have moderate predictive power at the decadal scale

In addition to determining whether genetic and demographic patterns are correlated, a practical concern for both approaches to assessing and conserving populations is understanding whether and which metrics predict the fates of populations through time. Interestingly, most demographic parameters were uncorrelated with contemporary population density, although predicted probability of extinction and observed contemporary population density were significantly negatively correlated in the East population group (Figure 5). Within the East group, the direction of relationships between other demographic metrics (long‐term population growth rates and predicted population size) and contemporary density were positive, but fairly weak. This suggests that in this case, as has been documented previously, demographic models that may accurately describe dynamics during the census period may still fail to precisely predict future population status, especially over longer spans of time (Crone et al., 2012). Conversely, such models may be sufficient for prediction in principle, but we were unable to detect relevant correlations in our small sample of populations, which substantially limits statistical power.

A major component limiting demographic prediction is uncertainty in how environmental conditions have changed at these sites over time between the census period and the present. Modeling environmental conditions as annual IPM kernels that are independently and identically distributed is reasonable when lacking a detailed understanding of which aspects of environmental variation specifically drive vital rates, and how those aspects have changed over time. However, this fails to account for biologically relevant sequences of environmental variation and changes in the means and variances of environmental drivers of demography over time. Similarly, the shapes of vital rate functions that drive population growth may themselves change over time (due to evolution and/or plasticity). Even when many years of demographic data are available, population projections must inherently make predictions about population growth from a limited set of observed environmental conditions. Limited sampling of environments is particularly problematic if populations experience nonlinear changes in vital rates at environmental extremes that may not be encountered during a demographic study, even one of moderate duration. This type of pattern essentially causes the shape of vital rate functions to “change” over time even in the absence of evolutionary change, since the ability to detect linear vs. nonlinear effects of environmental drivers on demography depends upon the range of environments sampled; relationships may seem linear under one range of environmental conditions, but nonlinear under a broader range.

Finally, the predictive utility of these demographic models may have also been limited because they did not incorporate density‐dependent dynamics. Particularly, if population growth showed negative density‐dependence (NDD), it would be expected to increase as populations decline in size, buffering against further decline. Population densities were quite high in some populations during the census period (Figure S6), suggesting that conspecific competition may have the potential to influence vital rates. The effects of competition with conspecifics are not known in this species, although there is some evidence that heterospecific competition can reduce *B. fecunda* fitness (Lesica & Shelly, 1992). Even in the absence of direct resource competition among adults, patterns of NDD may arise in populations if availability of suitable habitat for new recruits is limited by existing established conspecifics. Because *B. fecunda* has a limited distribution on a specific substrate (Lesica, 1993), this mechanism of NDD is also plausible in this species. Further research into the density‐dependent dynamics of this species may improve the performance of demographic models.

### 4.3 Genetic parameters are not correlated with contemporary population density

Neither observed heterozygosity nor the inbreeding coefficient was significantly correlated with contemporary population densities (Figure 5), although there was a weak trend suggesting that populations with higher observed heterozygosity in the 1990s have higher densities at present. As with the demographic patterns described above, this relationship is only apparent in the East population group. If this relationship is real, either direction of causality is plausible; more genetically diverse populations may have been able to achieve or maintain relatively higher densities over time (although still declining; Figure S7) because of that genetic variation (e.g., through adaptation; Lande & Shannon, 1996), but populations that are larger may also simply harbor greater standing variation because it has not yet been lost through population bottlenecks, stochastic demographic events, etc. Such ambiguity of causality is common in studies considering the relationships among genetics and demography, however, and such distinctions may not be particularly meaningful; evolutionary change to the genetic composition of populations is, by definition, both a cause and a consequence of demographic variation (Retel et al., 2019). Nevertheless, positive relationships between genetic variation and contemporary population size would raise the possibility that adaptation over time may have contributed to persistence of these populations from the 1990s to the present. However, we also emphasize, as others have previously (Reed, 2010; Reed & Frankham, 2001, and references therein), that surveys of neutral genetic variation are not necessarily correlated with “adaptive potential”, *per se*, and caution against the interpretation of it as such in absence of data on genetic variation underlying adaptive traits.

Interestingly, as above, the inbreeding coefficient was not even weakly correlated with contemporary population densities in *B. fecunda* (Figure 5, lower right). Because much work focuses on ID as a correlate of genetic variation and population performance, metrics of genetic variation other than *F*_IS_ are less commonly assessed in relation to demographic dynamics (but see Endels et al., 2007; Richards et al., 2003). Some of this emphasis on *F*_IS_ may also be driven by the implicit assumption that more inbred populations have lower standing genetic variation. In this case, because *B. fecunda* is self‐pollinating but not particularly depauperate of genetic variation compared with widespread relatives (Song & Mitchell‐Olds, 2007), *F*_IS_ is not negatively correlated with other metrics of genetic variation. This provides the insight that, while standing genetic variation may be beneficial for population viability, inbreeding does not necessarily mediate that relationship.

### 4.4 Conservation implications

Our findings suggest that increased genetic variation may correlate with more favorable population dynamics in *B. fecunda* (Figure 4). Thus, transplantation or translocation of individuals among populations to increase genetic variation within populations, as has been recommended for management of some other rare species (Luijten et al., 2002), may improve population viability and/or adaptive potential in this species. However, such action may also increase outcrossing. In the absence of evidence that ID influences population dynamics, it is not clear that inbreeding necessarily has negative effects on the population viability of *B. fecunda*. The converse implication is that more outbred populations do not appear to be more stable or viable than inbred ones. Results from this study do not support deliberate crossing among populations for managed reintroductions, as has sometimes been advocated in other systems (Frankham, 2015; Maschinski et al., 2013; Vilas et al., 2006). We emphasize that the potential benefits of translocations must be weighed against the potential risks of disrupting patterns of local adaptation (Leamy et al., 2014) by possibly promoting outcrossing. In this case, because populations are still extant despite predicted declines, we recommend further study investigating the extent of local adaptation in this species, if any, and the environmental drivers of adaptation. Such data would enable more tailored conservation recommendations and lend a mechanistic understanding to the relationship between genetic variation and demographic performance. In the absence of such data, protection of distinct, possibly locally adapted genetic variation within populations, as has been recommended previously in this species (Leamy et al., 2014; Song & Mitchell‐Olds, 2007), may be the most conservative approach until more is known.

In general, this study exemplifies the difficulty of making management recommendations based on assumed relationships between genetic variation and population viability. Whether the baseline assumption is that genetic variation indicates robust populations that are likely to persist over time or that a history of self‐fertilization may dampen the effects of ID on fitness, it is clear that many aspects of a species' natural history (e.g., mating system), environment (e.g., stressful vs. permissive conditions, connectivity to other populations), and evolutionary history (e.g., population bottlenecks, past purging) interact to inform and complicate relationships between genetics and population performance. Thus, in applying these results to the conservation of other species, we emphasize that, while biological links between genetic variation and demographic performance indeed exist, the magnitude and direction of those relationships, and their stability over time, are not always straightforward to predict. Thus, we recommend the collection of empirical data capturing both demographic trends and genetic variation in rare populations whenever feasible.

### 4.5 Caveats and limitations

While the demographic and genetic data reported here were collected contemporaneously in the 1990s, our ability to assess correlations between genetics and demography are constrained by a single snapshot view of genetic variation in these populations. Because genetic variation itself is likely to change over time as populations evolve and population sizes fluctuate, focusing on one‐time measurements of genetic variation is inherently limiting. However, prior studies tracking population genetic metrics over time have shown that some genetic parameters (including number of alleles and heterozygosity) may remain fairly constant over time (Noël et al., 2010; Richards et al., 2003). Similarly, on the decadal scale in another highly selfing species, the degree of change in population genetic parameters over time was found to be markedly less than the variation observed across space (Gomaa et al., 2011). Thus, using snapshot metrics of genetic variation is limited, but may still capture variation that is relevant, at least on the scale of decades. Further, this limitation is not unique; of the comparably few studies explicitly relating genetics to population performance, very few characterize genetic variation at more than one or a few points in time, and even data testing for transient relationships in the near term are lacking (Endels et al., 2007; Gomaa et al., 2011; Richards et al., 2003). Regardless, in the future, we recommend additional monitoring of population genetic variation in *B. fecunda* populations, as changes in genetic variation over time may be better predictors of demographic trends than fixed estimates (Gomaa et al., 2011; Richards et al., 2003).

Our inference about genetic and demographic relationships is also strongly limited by sample size and statistical power; while our demographic data track the survival, reproduction, and recruitment of ~18,000 individual plants, testing for population–level trends reduces the level of replication to just five populations. To have moderate power of detection (0.75) of statistical significance at *α* = 0.05, the strength of relationships must be substantial (|*r*| > 0.94) when the sample size is 5 (Champley, 2020; Cohen, 1988). Of course, this threshold becomes even more stringent when focusing on a subset of the populations or adjusting α to correct for multiple testing (which, in this case, we chose not to do since most population–level relationships were already weak or nonsignificant at the more permissive *α* = 0.05 level). Despite this limited power, some tested relationships still showed significant or marginally significant correlations (Figure 4), even when focusing solely on the East population group (Figure 5).

Finally, because we relied on previously published population genetic summary statistics, we could not account for the effect of uncertainty in estimates of genetic variation, which may reduce the power to detect relationships between genetic variation and demographic trends (Bozzuto et al., 2019). Despite this, very few studies have yet been able to explicitly test for relationships between genetic patterns and demographic processes; thus, in a subfield lacking datasets spanning both demographic monitoring over time and measurements of genetic variation, our results still represent an advance.

### 4.6 Future directions

In general, we advocate for further studies investigating the roles of genetic variation and inbreeding in population persistence. As genetic and genomic sequencing technologies become increasingly affordable and tractable in nonmodel species, we hope that genetic variation and its changes over time will be assessed in a greater number of demographic studies. Presently, the lack of evidence for such population–level relationships may be due in part to a lack of sufficient data rather than to the absence of biological links between genetics and demography. Thus far, the most robust test of population–level effects of genetic variation has been applied to reintroduced populations of the Alpine ibex, a mammal species that previously suffered severe population bottlenecks (Bozzuto et al., 2019). Because of the complicating factors of ecological context, evolutionary history, and mating system, it is important to replicate such studies across species with variable life histories, including plants such as *B. fecunda* that have mixed mating systems. In this case, our findings that heterozygosity but not inbreeding *per se* is related to demographic performance support the expectation that species and populations with varying histories of inbreeding may not show ID at the population level (Table 1).

New analytical frameworks that explicitly account for genetic variation when modeling demography may broaden the ways in which such relationships can be quantified and prompt further integration of these subfields (de Vries & Caswell, 2019). Finally, rapid advancements in the scope and quality of genomic data will permit more nuanced tests of relationships between genetics and demographic performance. Recent work in other systems has identified alternative signatures of inbreeding in genomic data, such as identical by descent runs of homozygosity (Foote et al., 2021; Ochoa & Gibbs, 2021), which (unlike *F*_IS_) are agnostic to Hardy–Weinberg assumptions. For *B. fecunda* specifically, the recent assembly and annotation of reference genomes for both East and West genetic groups (Zhang et al., 2020) provides a new resource to explore genetic variation in functional as well as neutral regions of the genome, and to assess the genetic basis of differentiation between populations and its contribution to demographic variation.

Finally, in addition to caveats such as changing environments discussed above, one factor that may frequently dampen the predictive power of demographic models is evolution in the focal populations over time. Over time, as genetic variation and/or traits that contribute to adaptation change, they may cause subsequent changes to population dynamics. For example, extinction risk in some populations may be buffered by the process of adaptation (i.e., experience evolutionary rescue; Bell, 2017). In this case, the persistence of all focal populations despite high likelihoods of extinction suggests that population dynamics have improved between 1997 and present. Evolution is certainly one process that could cause this change. Future studies explicitly interrogating change in genetic and/or phenotypic variation over time in these populations, such as resurrection studies (Hamman et al., 2018), may reveal the magnitude of adaptive (or maladaptive) evolutionary change that has occurred in recent decades.

## CONFLICT OF INTEREST

The authors declare no competing interests.

## BENEFIT‐SHARING STATEMENT

This research was executed in consultation with the Montana Natural Heritage Program, and the data and results described in this manuscript were shared with program botanists to facilitate monitoring and management of the focal species. Benefits from this research also accrue from the sharing of our data and results on public databases, as described above.

## Supporting information


Appendix S1

## Data Availability

Data and code associated with this article have been uploaded to the Dryad Digital Repository under https://doi.org/10.5061/dryad.k98sf7m70 (Carley et al., 2022).

## APPENDIX A DETAILED DEMOGRAPHIC MODELING METHODS

### Data filtering

Four observations in the demographic dataset (<<1%) were extreme outliers for size (rosette diameter >100 mm; mean rosette diameter: 11.9 mm). We excluded these four observations from analyses in which rosette size was a predictor or response variable.

### Vital rate regressions

#### State transitions

We used mixed‐effects logistic regressions with binomial error to model five size‐dependent state transitions (Table S1A): the probability of becoming dormant, the probability of exiting dormancy, the probability of a dormant plant becoming a bolter, the probability of a “normal” plant becoming a bolter, and the probability of a bolter remaining a bolter. Since dormant plants have no aboveground tissue and bolters have no basal rosette, rosette diameter prior to dormancy and prior to becoming a bolter were used as the independent size variables, respectively, for those state transitions. We then modeled other vital rates (survival, growth, and reproduction) specifically within each of these discrete states (below; Figure S1B).

#### Survival

We used mixed‐effects logistic regressions with binomial error to model size‐dependent survival to year *t +* 1 of bolters and nonbolters (Table S1B). For nonbolters, rosette diameter in year *t* was the independent variable. For bolters, rosette diameter prior to becoming a bolter was the independent variable.

#### Growth

We fit growth functions specific to each discrete state in *B. fecunda's* life cycle (Table S1C; Figure S1B). For “normal” plants that were neither dormant nor bolters in either year *t* or year *t* + 1, we modeled growth by predicting mean size in year *t +* 1 from size in year *t* using linear mixed‐effects regressions. We accommodated size‐dependent variance in growth by modeling the variance around the mean size in year *t +* 1 as also potentially dependent on size in year *t*.

For plants that were exiting dormancy, we modeled mean size in year *t* + 1 as dependent on predormancy size using linear mixed‐effects regressions. We modeled the variance around mean size in year *t* + 1 as the residual variance in the model predicting mean size, following model selection.

For plants that ceased being bolters and regrew rosettes, we modeled mean size in year *t* + 1 as dependent on prebolter size using linear mixed‐effects regressions. We modeled the variance around mean size in year t + 1 as the residual variance in the best‐fit model predicting mean size, following model selection.

Because individuals that became bolters or dormant had size 0 or no size, respectively, we did not model growth in these state spaces. In the multimatrix IPM approach (see below), we applied the identity matrix to these state spaces to retain prebolting or predormancy size measurements across projection time steps, since future size‐dependent vital rates and state transitions may depend on past sizes.

#### Reproduction

Because reproduction data were zero‐inflated, we partitioned total reproductive output into two models: a mixed‐effects logistic regression with binomial error for the probability of reproducing, and a generalized linear mixed‐effects model with a Poisson distribution for the number of fruits produced. For the former, we filtered data to include only individuals that were alive in both time *t* and time *t* + 1, *i.e*., modeling the probability of reproduction conditional upon survival. For the latter, we filtered data to include only living individuals that successfully reproduced, *i.e*., modeling fruit number conditional upon both survival and reproduction. We fit separate models for nondormant nonbolter individuals and nondormant bolters (Table S1D). Because in any one year and population combination there were relatively few bolters, data were pooled across all 5 populations for both reproduction functions. Because dormant individuals (regardless of past state) had no aboveground biomass and cannot reproduce, we assigned reproductive values of 0 to all dormant state spaces in the multimatrix IPM (see below).

#### Recruitment

While individual plants were tracked longitudinally across years, the parentage of new recruits in each population was not possible to assess. Because of this, we modeled two additional vital rate functions as independent of parental size: recruitment of new individuals into the population, and the size distribution of new recruits (Table S1E). To estimate the rate of recruitment of new individuals into each population, we summed the number of fruits and new recruits in each quadrat in each year, and modeled the total recruits at time *t* + 1 in response to the total fruits at time *t*, including random effects of transect and year. This recruitment rate encompasses the net effects of seed number per fruit, germination, and establishment of new germinants, none of which were directly measured in these censuses.

To determine the size distribution of new recruits, we modeled the mean log‐transformed rosette diameter of new recruits in each population and each year in response to random effects of year and quadrat nested within transect. We modeled the variance in the size of new recruits as the residual variance in these intercept‐only random effects models. We assigned new recruits projected by our demographic model a size value from a population‐ and year‐specific lognormal distribution defined by these empirical parameters. Because *B. fecunda* is not thought to have a seed bank (Lesica & Shelly, 1996) and because we lacked data to incorporate seed bank dynamics, we did not incorporate seed dormancy in our demographic models.

### Integral projection modeling

#### Construction of annual matrices: details on year effects

Because census year was included as a random effect in each model, we began by adding year‐specific deviations to the intercept for each vital rate function. To incorporate parameter uncertainty, we sampled from the distribution of the intercept's variance using a chi‐squared distribution. To sample the year‐specific random deviations from the intercept, we multiplied the variance across years estimated from the best‐fit model (following model selection) by a random draw from a chi‐squared distribution given the degrees of freedom for estimating the random effect of year in each model. Because completely random draws of annual variation may reshuffle the rank order of year effects that may be biologically meaningful (e.g., reducing survival to a low level in a year that was actually a “good” year), we retained the rank order annual deviations in our sampling scheme using the empirical cumulative distribution function of the annual deviations from the best‐fit model. For any annual deviations that were not observed directly in the vital rate regressions, we used the mean of the sampled values for all observed years.

#### Starting size distribution for *λ*_S_ calculations

Within each bootstrap run, we began projections with a vector of individuals distributed across IPM meshpoint classes using a uniform distribution. We distributed the total number of individuals in each population in 1997 across the three discrete states according to the proportion of individuals in each state at the stable age distribution, which we calculated from the mean multimatrix kernel across all census years. We renormalized the sum of the resultant vector to 1 after each projection step.

#### Starting size distribution for population projections

While estimating transient population dynamics in which outcomes are more sensitive to starting size distributions, we started projections using a Weibull distribution of sizes rather than a uniform distribution. We chose the Weibull distribution as the best descriptor of size distributions in 1997 (the starting point for forward projections) after qualitatively and quantitatively comparing goodness of fit of four candidate distributions (Weibull, log‐normal, gamma, and exponential; Figure S8) using the package ‘fitdistrplus’ in R (Delignette‐Muller & Dutang, 2015).

We then estimated Weibull parameters (shape and scale) to describe the distribution of sizes in each of the five populations in 1997. We initialized the starting size vector for projections by multiplying the vector of IPM meshpoint sizes by the empirical Weibull distribution as defined by each population‐specific set of estimated parameters. To scale individuals across discrete states, which could not be inferred in the final year of field censusing, we divided the total population size across the three discrete states according to the proportion of individuals in each state at the stable age distribution (as described above). In other words, the sum of the starting size vector was equal to the empirical population size in 1997, with the number of individuals in each discrete state determined by proportion of individuals occupying each in the stable size distribution, and distributed across a population‐specific Weibull distribution within each state space.
