# Supplementary material for: Are genetic variation and demographic performance linked?
Source: Evol Appl. 2022 Oct 4;15(11):1888–906. doi: 10.1111/eva.13487 (PMC9679243; doi:10.1111/eva.13487)
Supplement: Supplementary file 1 — Appendix S1 [file EVA-15-1888-s001.pdf]

Supplementary Information

Supplementary Figures

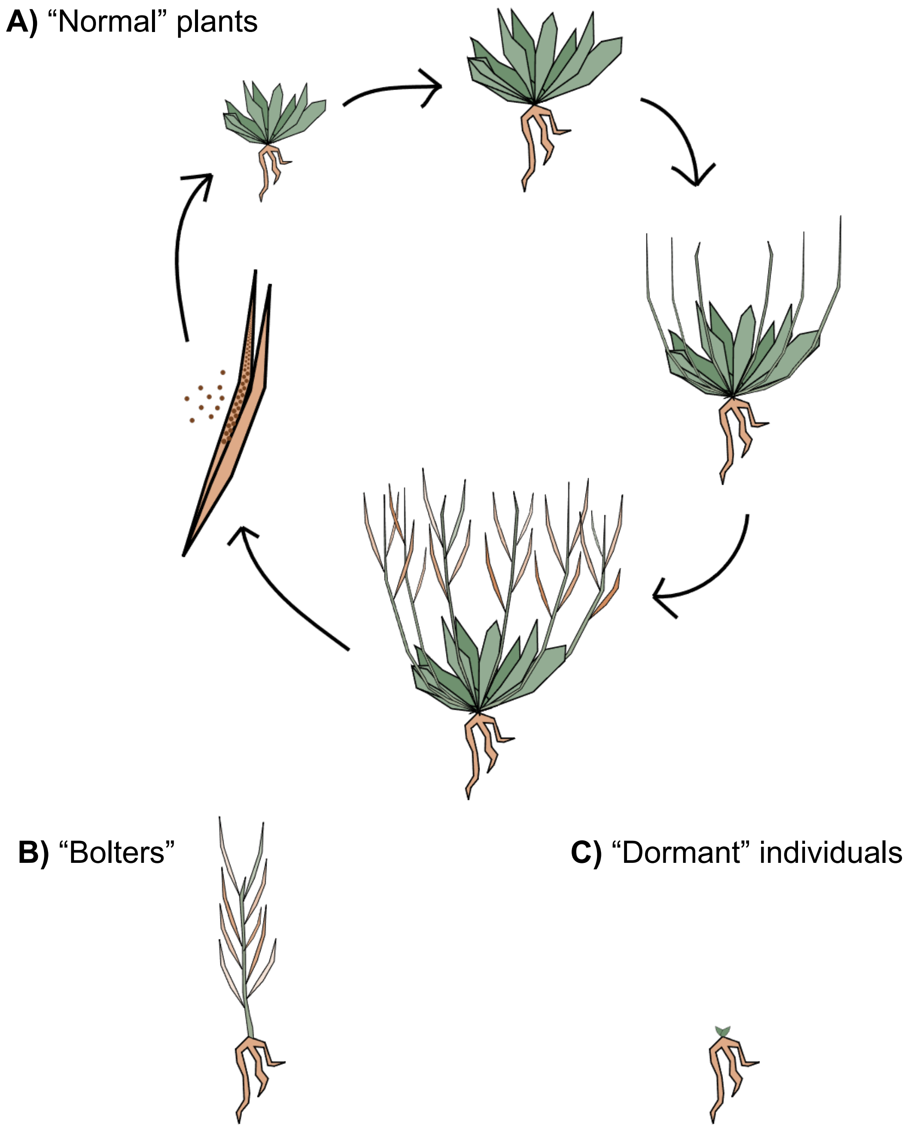

Figure S1. *B. fecunda* life-history. **A:** The life cycle of a “normal” plant involves vegetative growth of the perennial basal rosette and semelparous or iteroparous reproduction on bolting stalks that emerge from axillary buds. **B:** “Bolters” may instead reproduce via a single bolting stalk from the terminal bud with little to no basal rosette tissue remaining. **C:** “Dormant” individuals may survive belowground even when aboveground tissue has died back. **Note:** Across years, “normal” plants may become dormant or become bolters, dormant plants may re-emerge as “normal” or as bolters, etc. For simplicity’s sake, all such possible arrows on the life-cycle diagram are not shown.

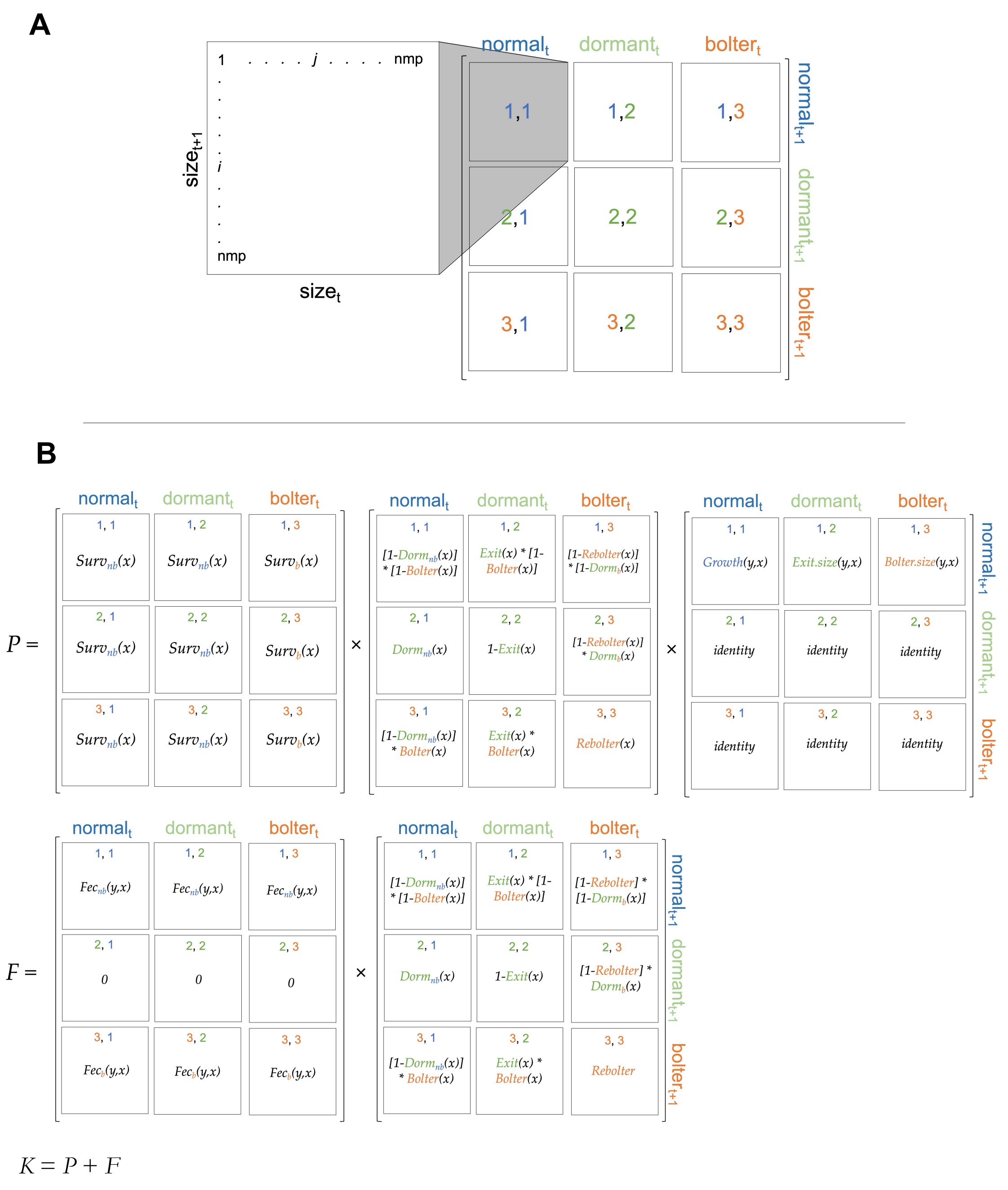

Figure S2. Graphical model of the multi-matrix IPM. **A:** General layout of $nmp \times nmp$ sub-matrices replicated across three discrete state spaces at times t, t+1. **B:** Applying state-specific survival, growth, and reproduction vital rate functions to sub-matrices accounts for biological variation in demographic rates at different life stages. Scaling both the growth/survival kernel (P) and the fecundity kernel (F) by a state-space transition multi-matrix accounts for the movement of individuals from one state to another across time. Full annual kernels (K) were constructed as the sum of the P and F multi-matrices.

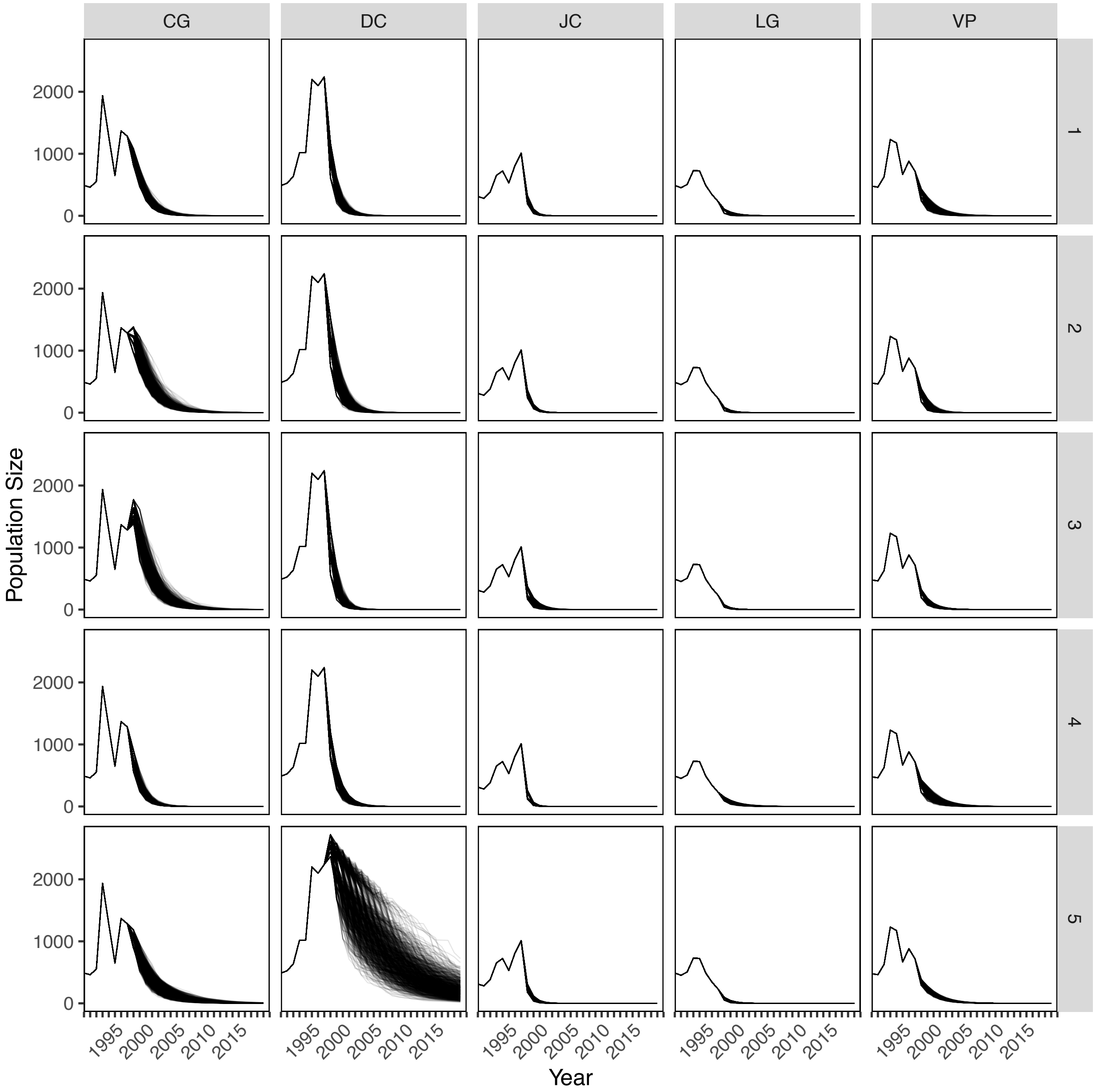

Figure S3. Examples of ”raw” population trajectories from which median population sizes were calculated to plot in Figure 3. Each column of panes shows the empirical (1990-1997) and predicted (1998-2019) population sizes for one of the focal populations. Rows of panes represent a randomly selected bootstrap run, in which vital rate regression coefficients were uniquely sampled from their distributions. Overlaid lines within each pane show 1,000 replicate projections within a bootstrap run. The median projected population size at each time step was calculated across the 1,000 replicate projections in each bootstrap run.

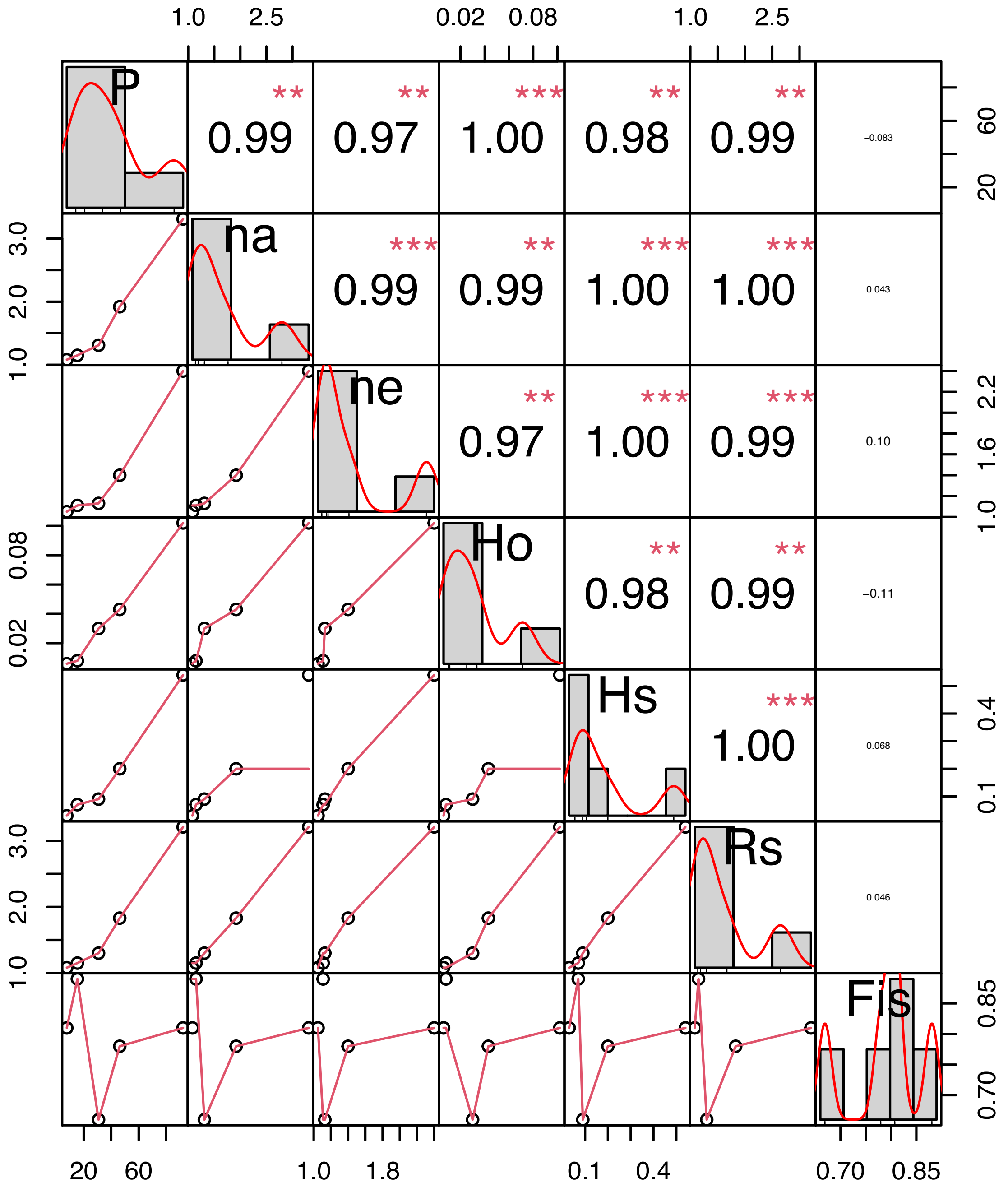

Figure S4. Across-population pairwise correlations among genetic data reported in Song & Mitchell-Olds (2007). On the diagonal, histograms (gray) and density plots (red) of each genetic parameter are shown, with the parameter name overlaid. Above the diagonal are correlation coefficients for all pairwise comparisons of parameters, with text size and asterisks indicating statistical significance. Below the diagonal are scatterplots of all pairwise comparisons, with lines connecting consecutive points.

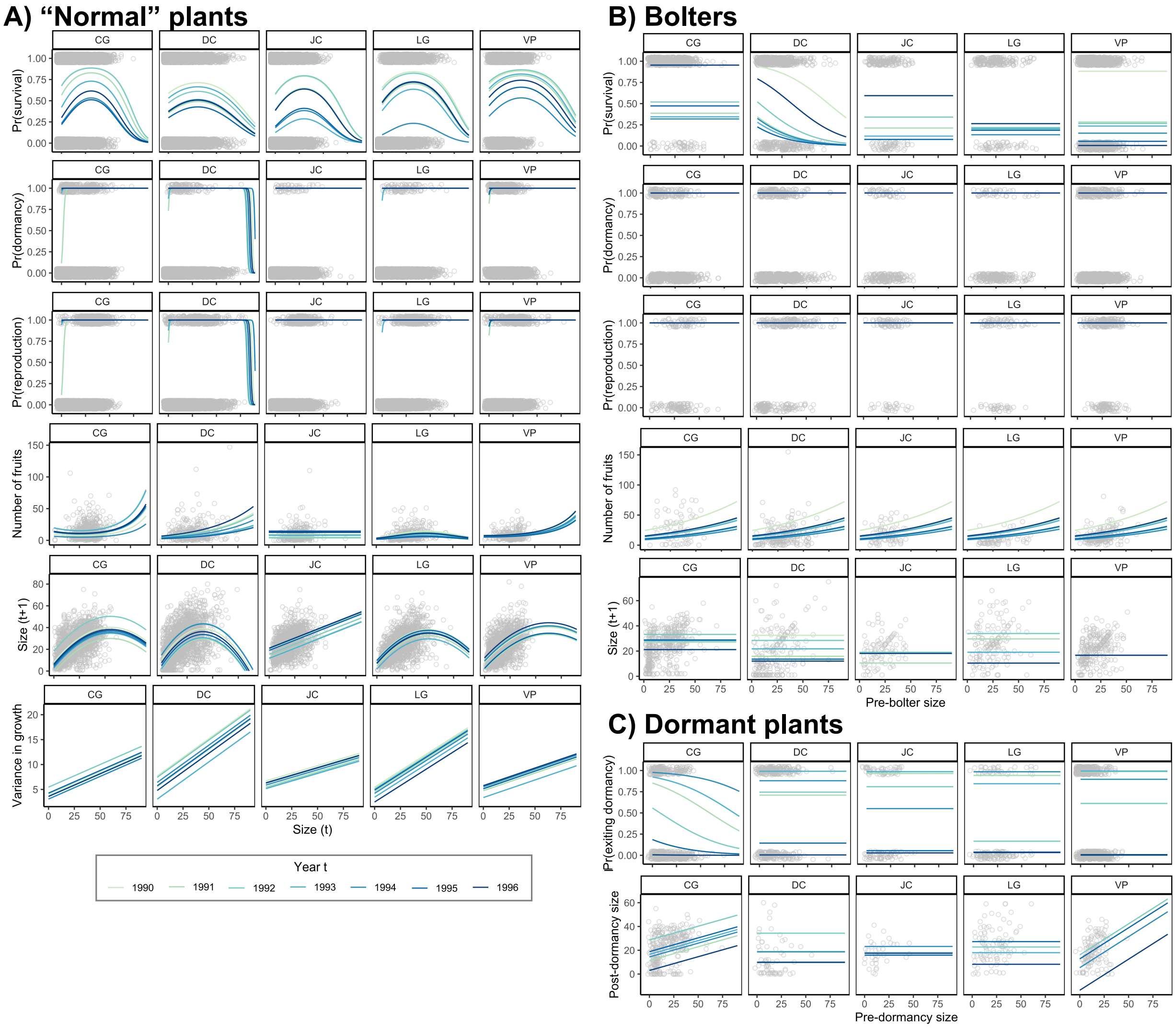

Figure S5. State-specific, size-dependent vital rate functions for five populations of *Boechera fecunda*: (**a**) non-dormant, non-bolter plants; (**b**) bolters, (**c**) dormant plants. Within each section, columns represent populations; CG = Charley's Gulch; DC = Dewey Cemetery; JC = Jerry Creek; LG = Lime Gulch; VP = Vipond Park. In each pane, gray points represent raw data, and lines represent predictions of best-fit vital rate regressions following model selection (back-transformed to the data scale in models using link functions). For all panes showing binary response variables, raw data points are jittered to reduce overlap. Raw data are not shown for size-dependent variance in growth (row 6 of **a**); models were fit using the absolute value of the residuals from each growth model (row 5 of **a**) as the response variable. Full details regarding model fitting and model selection are provided in Appendix 1.

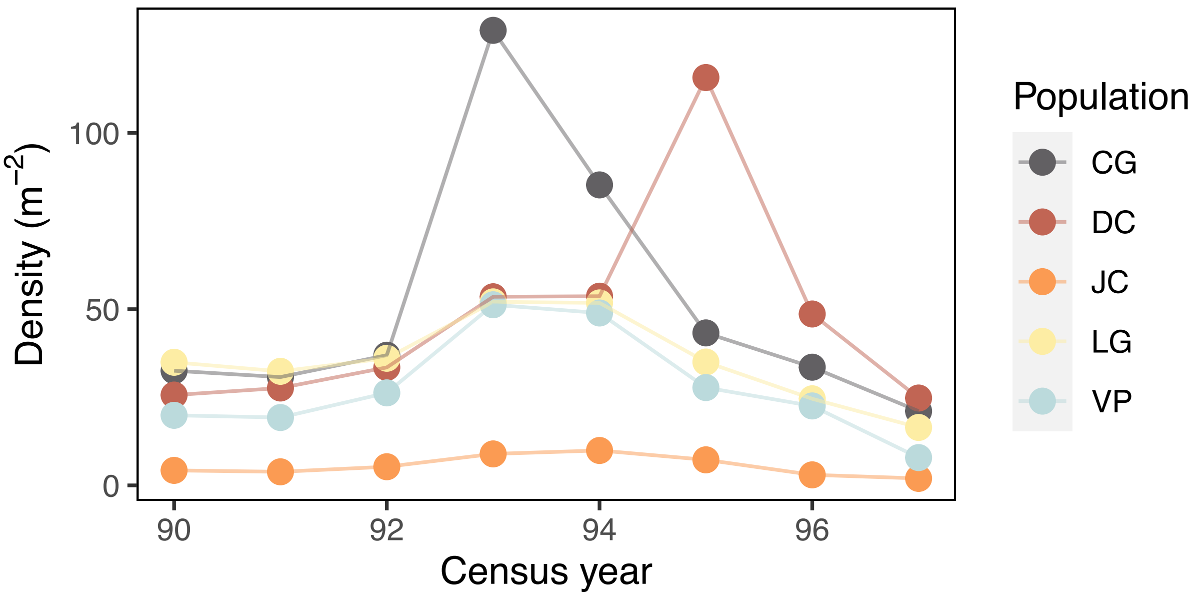

Figure S6. Observed population density (inferred from census population size divided by the number of quadrats searched per population) during historical censuses from 1990-1997.

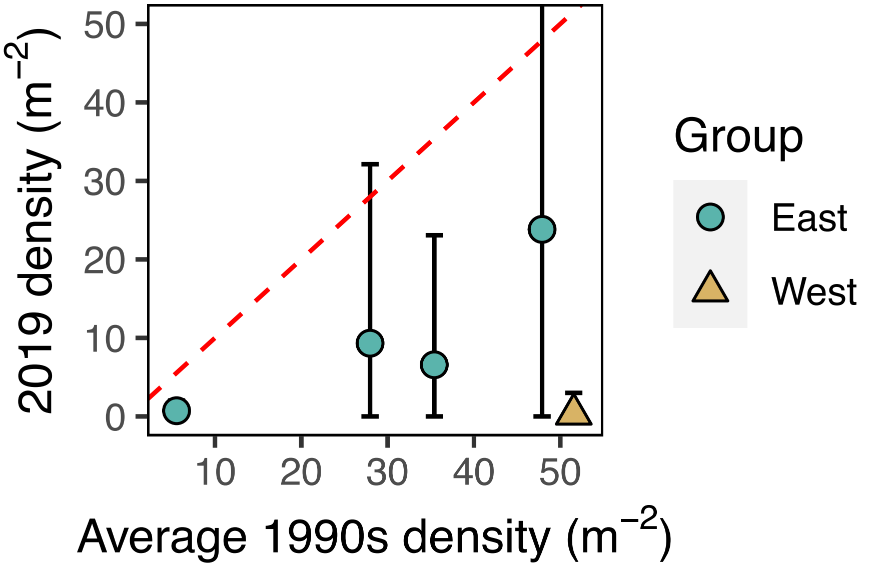

Figure S7. Comparison of mean density of each focal population across the 1990s census years and the contemporary density measured during the 2019 re-census. Points represent mean values, and the vertical error bars denote the 95% confidence interval (CI) for the estimate. For clarity, the y-axis sis truncated at 50, although the CIs for one population exceed this density. The red dashed line is the 1:1 line, indicating no change in population density between the 1990s and present.

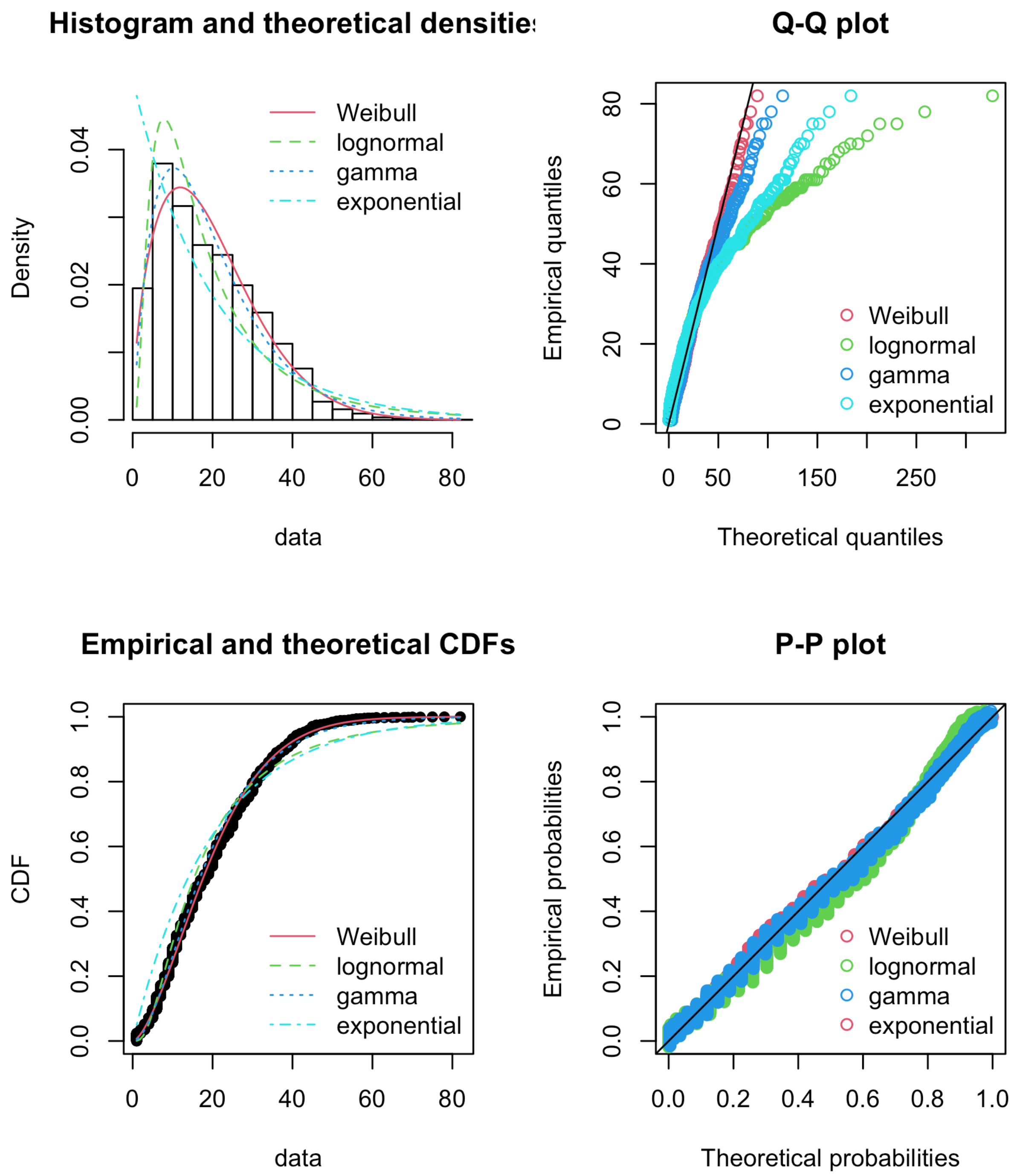

Figure S8. Comparisons of fit between empirical and theoretical distributions (Weibull, lognormal, gamma, and exponential) of new recruit size, using the raw data (top left), quantiles (top right), cumulative distribution functions (bottom left), and probabilities (bottom right). Empirical distributions are depicted in black, and the color of each tested theoretical distribution is indicated within the panes.

Supplementary Tables

Table S1. The form of best-fit vital rate regressions (VRRs) retained following model selection. **A:** State transitions. **B:** State-specific functions influencing survival. **C:** State-specific functions influencing growth. **D:** State-specific functions influencing reproduction. **E:** Recruitment. Growth, size upon exiting dormancy, and log recruit size were modeled using linear mixed models (LMMs), which assume Normally distributed errors. All generalized linear mixed effects models (GLMMs) have a binomial error distribution unless otherwise noted. In general, the response variable for each individual i in year j and located in quadrat k nested within transect l is modeled as a fixed intercept (β_0) modified by random effects of year ($year_j$) and location ($quad_{k,l}$), optional linear ($\beta_1 \times size_{i,j,k,l}$) and/or quadratic ($\beta_2 \times \ size^2{}_{i,j,k,l}$) effects of size (depending on the outcome of model selection), and error ($\varepsilon_{i,j,k,l} \sim Normal(0, \sigma^2)$, where σ^2 is the residual variance, unless otherwise noted). For any vital rates modeled in context of a state change in which there is no size measurement in year j, time steps before and after the state change are indicated as *j_pre* and *j_post* instead of *j* and *j+1*, respectively, as appropriate.

Component	VRR (form)	Pop.	Best-fit model
**(A)** State transitions	Probability of becoming a bolter – non-bolters (binomial GLMM)	CG	$bolter_{i,j+1,k,l} = \beta_0 + year_j + quad_{k,l} + \beta_1 \times size_{i,j,k,l} + \varepsilon_{i,j,k,l}$
		DC	$bolter_{i,j+1,k,l} = \beta_0 + year_j + quad_{k,l} + \beta_1 \times size_{i,j,k,l} + \varepsilon_{i,j,k,l}$
		JC	$bolter_{i,j+1,k,l} = \beta_0 + year_j + quad_{k,l} + \beta_1 \times size_{i,j,k,l} + \varepsilon_{i,j,k,l}$
		LG	$bolter_{i,j+1,k,l} = \beta_0 + year_j + quad_{k,l} + \beta_1 \times size_{i,j,k,l} + \varepsilon_{i,j,k,l}$
		VP	$bolter_{i,j+1,k,l} = \beta_0 + year_j + quad_{k,l} + \beta_2 \times size^2{}_{i,j,k,l} + \varepsilon_{i,j,k,l}$
	Probability of remaining a bolter – bolters (binomial GLMM)	CG	$bolter_{i,j\_post,k,l} = \beta_0 + year_j + quad_{k,l} + \beta_1 \times size_{i,j\_pre,k,l} + \varepsilon_{i,j,k,l}$
		DC	$bolter_{i,j\_post,k,l} = \beta_0 + year_j + quad_{k,l} + \beta_1 \times size_{i,j\_pre,k,l} + \varepsilon_{i,j,k,l}$
		JC	$bolter_{i,j\_post,k,l} = \beta_0 + year_j + quad_{k,l} + \beta_1 \times size_{i,j\_pre,k,l} + \varepsilon_{i,j,k,l}$
		LG	$bolter_{i,j\_post,k,l} = \beta_0 + year_j + quad_{k,l} + \beta_1 \times size_{i,j\_pre,k,l} + \beta_2 \times size^2{}_{i,j\_pre,k,l} + \varepsilon_{i,j,k,l}$
		VP	$bolter_{i,j\_post,k,l} = \beta_0 + year_j + quad_{k,l} + \beta_1 \times size_{i,j\_pre,k,l} + \beta_2 \times size^2{}_{i,j\_pre,k,l} + \varepsilon_{i,j,kl}$
	Probability of entering dormancy – bolters (binomial GLMM)	CG	$dormant_{i,j+1,k,l} = \beta_0 + year_j + quad_{k,l} + \beta_1 \times size_{i,j\_pre,k,l} + \varepsilon_{i,j,k,l}$
		DC	$dormant_{i,j+1,k,l} = \beta_0 + year_j + quad_{k,l} + \beta_1 \times size_{i,j\_pre,k,l} + \varepsilon_{i,j,k,l}$
		JC	$dormant_{i,j+1,k,l} = \beta_0 + year_j + quad_{k,l} + \beta_1 \times size_{i,j\_pre,k,l} + \varepsilon_{i,j,k,l}$
		LG	$dormant_{i,j+1,k,l} = \beta_0 + year_j + quad_{k,l} + \varepsilon_{i,j,k,l}$
		VP	$dormant_{i,j+1,k,l} = \beta_0 + year_j + quad_{k,l} + \beta_1 \times size_{i,j\_pre,k,l} + \beta_2 \times size^2{}_{i,j\_pre,k,l} + \varepsilon_{i,j,k,l}$

	Probability of entering dormancy – non-bolters (binomial GLMM)	CG	$dormant_{i,j+1,k,l} = \beta_0 + year_j + quad_{k,l} + \beta_1 \times size_{i,j,k,l} + \varepsilon_{i,j,k,l}$
		DC	$dormant_{i,j+1,k,l} = \beta_0 + year_j + quad_{k,l} + \varepsilon_{i,j,k,l}$
		JC	$dormant_{i,j+1,k,l} = \beta_0 + year_j + quad_{k,l} + \varepsilon_{i,j,k,l}$
		LG	$dormant_{i,j+1,k,l} = \beta_0 + year_j + quad_{k,l} + \varepsilon_{i,j,k,l}$
		VP	$dormant_{i,j+1,k,l} = \beta_0 + year_j + quad_{k,l} + \beta_1 \times size_{i,j,k,l} + \varepsilon_{i,j,k,l}$
	Probability of exiting dormancy (binomial GLMM)	CG	$dormant_{i,j+1,k,l} = \beta_0 + year_j + quad_{k,l} + \beta_1 \times size_{i,j\_pre,k,l} + \varepsilon_{i,j,k,l}$
		DC	$dormant_{i,j+1,k,l} = \beta_0 + year_j + quad_{k,l} + \varepsilon_{i,j,k,l}$
		JC	$dormant_{i,j+1,k,l} = \beta_0 + year_j + quad_{k,l} + \varepsilon_{i,j,k,l}$
		LG	$dormant_{i,j+1,k,l} = \beta_0 + year_j + quad_{k,l} + \varepsilon_{i,j,k,l}$
		VP	$dormant_{i,j+1,k,l} = \beta_0 + year_j + quad_{k,l} + \varepsilon_{i,j,k,l}$
**(B)** Survival	Probability of survival – bolters (binomial GLMM)	CG	$survival_{i,j+1,k,l} = \beta_0 + year_j + quad_{k,l} + \varepsilon_{i,j,k,l}$
		DC	$survival_{i,j+1,k,l} = \beta_0 + year_j + quad_{k,l} + \beta_1 \times size_{i,j\_pre,k,l} + \varepsilon_{i,j,k,l}$
		JC	$survival_{i,j+1,k,l} = \beta_0 + year_j + quad_{k,l} + \varepsilon_{i,j,k,l}$
		LG	$survival_{i,j+1,k,l} = \beta_0 + year_j + quad_{k,l} + \varepsilon_{i,j,k,l}$
		VP	$survival_{i,j+1,k,l} = \beta_0 + year_j + quad_{k,l} + \varepsilon_{i,j,k,l}$
	Probability of survival – non-bolters (binomial GLMM)	CG	$survival_{i,j+1,k,l} = \beta_0 + year_j + quad_{k,l} + \beta_1 \times size_{i,j,k,l} + \beta_2 \times size^2{}_{i,j,k,l} + \varepsilon_{i,j,k,l}$
		DC	$survival_{i,j+1,k,l} = \beta_0 + year_j + quad_{k,l} + \beta_1 \times size_{i,j,k,l} + \beta_2 \times size^2{}_{i,j,k,l} + \varepsilon_{i,j,k,l}$
		JC	$survival_{i,j+1,k,l} = \beta_0 + year_j + quad_{k,l} + \beta_1 \times size_{i,j,k,l} + \beta_2 \times size^2{}_{i,j,k,l} + \varepsilon_{i,j,k,l}$
		LG	$survival_{i,j+1,k,l} = \beta_0 + year_j + quad_{k,l} + \beta_1 \times size_{i,j,k,l} + \beta_2 \times size^2{}_{i,j,k,l} + \varepsilon_{i,j,k,l}$
		VP	$survival_{i,j+1,k,l} = \beta_0 + year_j + quad_{k,l} + \beta_1 \times size_{i,j,k,l} + \beta_2 \times size^2{}_{i,j,k,l} + \varepsilon_{i,j,k,l}$
**(C)** Growth	Mean growth – bolters (LMM)	CG	$size_{i,j+1,k,l} = \beta_0 + year_j + quad_{k,l} + \varepsilon_{i,j,k,l}$
		DC	$size_{i,j+1,k,l} = \beta_0 + year_j + quad_{k,l} + \varepsilon_{i,j,k,l}$
		JC	$size_{i,j+1,k,l} = \beta_0 + year_j + quad_{k,l} + \varepsilon_{i,j,k,l}$
		LG	$size_{i,j+1,k,l} = \beta_0 + year_j + quad_{k,l} + \varepsilon_{i,j,k,l}$
		VP	$size_{i,j+1,k,l} = \beta_0 + year_j + quad_{k,l} + \varepsilon_{i,j,k,l}$
	Mean growth – non-bolters (LMM)	CG	$size_{i,j+1,k,l} = \beta_0 + year_j + quad_{k,l} + \beta_1 \times size_{i,j,k,l} + \beta_2 \times size^2{}_{i,j,k,l} + \varepsilon_{i,j,k,l}$
		DC	$size_{i,j+1,k,l} = \beta_0 + year_j + quad_{k,l} + \beta_1 \times size_{i,j,k,l} + \beta_2 \times size^2{}_{i,j,k,l} + \varepsilon_{i,j,k,l}$
		JC	$size_{i,j+1,k,l} = \beta_0 + year_j + quad_{k,l} + \beta_1 \times size_{i,j,k,l} + \varepsilon_{i,j,k,l}$

		LG	$size_{i,j+1,k,l} = \beta_0 + year_j + quad_{k,l} + \beta_1 \times size_{i,j,k,l} + \beta_2 \times size^2{}_{i,j,k,l} + \varepsilon_{i,j,k,l}$
		VP	$size_{i,j+1,k,l} = \beta_0 + year_j + quad_{k,l} + \beta_1 \times size_{i,j,k,l} + \beta_2 \times size^2{}_{i,j,k,l} + \varepsilon_{i,j,k,l}$
	Variance in growth – non-bolters (LMM)	CG	$\lvert residuals\ of\ growth\ model\rvert_{i,j,k,l} = \beta_0 + year_j + quad_{k,l} + \beta_1 \times size_{i,j,k,l} + \varepsilon_{i,j,k,l}$
		DC	$\lvert residuals\ of\ growth\ model\rvert_{i,j,k,l} = \beta_0 + year_j + quad_{k,l} + \beta_1 \times size_{i,j,k,l} + \varepsilon_{i,j,k,l}$
		JC	$\lvert residuals\ of\ growth\ model\rvert_{i,j,k,l} = \beta_0 + year_j + quad_{k,l} + \beta_1 \times size_{i,j,k,l} + \varepsilon_{i,j,k,l}$
		LG	$\lvert residuals\ of\ growth\ model\rvert_{i,j,k,l} = \beta_0 + year_j + quad_{k,l} + \beta_1 \times size_{i,j,k,l} + \varepsilon_{i,j,k,l}$
		VP	$\lvert residuals\ of\ growth\ model\rvert_{i,j,k,l} = \beta_0 + year_j + quad_{k,l} + \beta_1 \times size_{i,j,k,l} + \varepsilon_{i,j,k,l}$
	Size upon exiting dormancy (LMM)	CG	$size_{i,j\_post,k,l} = \beta_0 + year_j + quad_{k,l} + \beta_1 \times size_{i,j\_pre,k,l} + \varepsilon_{i,j,k,l}$
		DC	$size_{i,j\_post,k,l} = \beta_0 + year_j + quad_{k,l} + \varepsilon_{i,j,k,l}$
		JC	$size_{i,j\_post,k,l} = \beta_0 + year_j + quad_{k,l} + \varepsilon_{i,j,k,l}$
		LG	$size_{i,j\_post,k,l} = \beta_0 + year_j + quad_{k,l} + \varepsilon_{i,j,k,l}$
		VP	$size_{i,j\_post,k,l} = \beta_0 + year_j + quad_{k,l} + \beta_1 \times size_{i,j\_pre,k,l} + \varepsilon_{i,j,k,l}$
**(D)** Reproduction	Probability of reproducing – bolters (binomial GLMM)	All	$reproduction_{i,j,k,l} = \beta_0 + year_j + quad_{k,l} + \varepsilon_{i,j,k,l}$
	Probability of reproducing – non-bolters (binomial GLMM)	CG	$reproduction_{i,j,k,l} = \beta_0 + year_j + quad_{k,l} + \beta_1 \times size_{i,j,k,l} + \beta_2 \times size^2{}_{i,j,k,l} + \varepsilon_{i,j,k,l}$
		DC	$reproduction_{i,j,k,l} = \beta_0 + year_j + quad_{k,l} + \beta_1 \times size_{i,j,k,l} + \beta_2 \times size^2{}_{i,j,k,l} + \varepsilon_{i,j,k,l}$
		JC	$reproduction_{i,j,k,l} = \beta_0 + year_j + quad_{k,l} + \beta_1 \times size_{i,j,k,l} + \beta_2 \times size^2{}_{i,j,k,l} + \varepsilon_{i,j,k,l}$
		LG	$reproduction_{i,j,k,l} = \beta_0 + year_j + quad_{k,l} + \beta_1 \times size_{i,j,k,l} + \beta_2 \times size^2{}_{i,j,k,l} + \varepsilon_{i,j,k,l}$
		VP	$reproduction_{i,j,k,l} = \beta_0 + year_j + quad_{k,l} + \beta_1 \times size_{i,j,k,l} + \beta_2 \times size^2{}_{i,j,k,l} + \varepsilon_{i,j,k,l}$
	Number of fruits produced – bolters (Poisson GLMM)	All	$fruits_{i,j} = \beta_0 + year_j + \beta_1 \times size_{i,j\_pre} + \varepsilon_{i,j}$
	Number of fruits produced – non-bolters (Poisson GLMM)	CG	$fruits_{i,j,k,l} = \beta_0 + year_j + quad_{k,l} + \beta_1 \times size_{i,j,k,l} + \beta_2 \times size^2{}_{i,j,k,l} + \varepsilon_{i,j,k,l}$
		DC	$fruits_{i,j,k,l} = \beta_0 + year_j + quad_{k,l} + \beta_1 \times size_{i,j,k,l} + \varepsilon_{i,j,k,l}$
		JC	$fruits_{i,j,k,l} = \beta_0 + year_j + quad_{k,l} + \varepsilon_{i,j,k,l}$
		LG	$fruits_{i,j,k,l} = \beta_0 + year_j + quad_{k,l} + \beta_1 \times size_{i,j,k,l} + \beta_2 \times size^2{}_{i,j,k,l} + \varepsilon_{i,j,k,l}$
		VP	$fruits_{i,j,k,l} = \beta_0 + year_j + quad_{k,l} + \beta_2 \times size^2{}_{i,j,k,l} + \varepsilon_{i,j,k,l}$

**(E)** Recruitment	Per quadrat recruitment rate (Poisson GLMM)	All	$recruits\ per\ quad_{j+1,k} = \beta_0 + year_j + \beta_1 \times fruits\ per\ quad_{j,k} + \varepsilon_{j,k}$
		DC	$recruits\ per\ quad_{j+1,k} = \beta_0 + year_j + \beta_1 \times fruits\ per\ quad_{j,k} + \varepsilon_{j,k}$
		JC	$recruits\ per\ quad_{j+1,k} = \beta_0 + year_j + \beta_1 \times fruits\ per\ quad_{j,k} + \varepsilon_{j,k}$
		LG	$recruits\ per\ quad_{j+1,k} = \beta_0 + year_j + \beta_1 \times fruits\ per\ quad_{j,k} + \varepsilon_{j,k}$
		VP	$recruits\ per\ quad_{j+1,k} = \beta_0 + year_j + \beta_1 \times fruits\ per\ quad_{j,k} + \varepsilon_{j,k}$
	Size of new recruits (LMM)	CG	$log\ (mean\ size\ per\ quadrat)_{i,j+1,k,l} = \beta_0 + year_j + quad_{k,l} + \varepsilon_{i,j,k,l}$
		DC	$log\ (mean\ size\ per\ quadrat)_{i,j+1,k,l} = \beta_0 + year_j + quad_{k,l} + \varepsilon_{i,j,k,l}$
		JC	$log\ (mean\ size\ per\ quadrat)_{i,j+1,k,l} = \beta_0 + year_j + quad_{k,l} + \varepsilon_{i,j,k,l}$
		LG	$log\ (mean\ size\ per\ quadrat)_{i,j+1,k,l} = \beta_0 + year_j + quad_{k,l} + \varepsilon_{i,j,k,l}$
		VP	$log\ (mean\ size\ per\ quadrat)_{i,j+1,k,l} = \beta_0 + year_j + quad_{k,l} + \varepsilon_{i,j,k,l}$
